# Effects of Acute and Chronic Exercises on Executive Function in Children and Adolescents: A Systemic Review and Meta-Analysis

**DOI:** 10.3389/fpsyg.2020.554915

**Published:** 2020-12-17

**Authors:** Shijie Liu, Qian Yu, Zaimin Li, Paolo Marcello Cunha, Yanjie Zhang, Zhaowei Kong, Wang Lin, Sitong Chen, Yujun Cai

**Affiliations:** ^1^School of Physical Education and Sport Training, Shanghai University of Sport, Shanghai, China; ^2^Exercise and Mental Health Laboratory, School of Psychology, Shenzhen University, Shenzhen, China; ^3^School of Wushu, Chengdu Sport University, Chengdu, China; ^4^Metabolism, Nutrition, and Exercise Laboratory, Londrina State University, Londrina, Brazil; ^5^Health and Exercise Science Laboratory, Institute of Sports Science, seoul National University, Seoul, South Korea; ^6^Faculty of Education, University of Macao, Macao, China; ^7^Department of Physical Education, Wuhan University of Technology, Wuhan, China

**Keywords:** executive function, acute exercise, chronic exercise, cognitive, adolescents

## Abstract

**Background:** Physical exercises can affect executive function both acutely and chronically, with different mechanisms for each moment. Currently, only a few reviews have elaborated on the premise that different types of exercises have different mechanisms for improving executive function. Therefore, the primary purpose of our systematic review was to analyze the effects of acute and chronic exercises on executive function in children and adolescents.

**Objective:** We identified acute and chronic exercise studies and randomized controlled trials (RCTs) of executive function in children and adolescents that reported overall effect, heterogeneity, and publication bias of acute and chronic exercises on executive function.

**Methods:** We searched for RCTs of exercise interventions in children and adolescents from databases including PubMed, Web of Science, Scopus, The Cochrane Library, CNKI (China National Knowledge Infrastructure), and Wanfang, from January 1 2009 to December 31 2019. We performed methodological quality evaluations on the included literature using the Physiotherapy Evidence Database Scale (PEDro) and graded evidence with a meta-analysis using Stata 12.0 software.

**Results:** In total, 36 RCTs were included (14 acute exercises, 22 chronic exercises); the overall results of the meta-analysis (4,577 students) indicated that acute exercises significantly improved working memory (standardized mean difference (SMD) = −0.72; 95% confidence interval (CI) −0.89 to −0.56; *p* < 0.001), inhibitory control (SMD = −0.25; 95% CI −0.40 to −0.09; *p* = 0.002), and cognitive flexibility (SMD = −0.34; 95% CI −0.55 to −0.14; *p* < 0.005), whereas chronic exercises significantly improved working memory (SMD = −0.54; 95% CI −0.74 to −0.33; *p* < 0.001), inhibitory control (SMD = −0.30; 95% CI −0.38 to −0.22; *p* < 0.001), and cognitive flexibility (SMD = −0.34, 95 % CI −0.48 to −0.20, *p* < 0.001).

**Conclusion:** Acute and chronic exercises can effectively improve the executive function of children and adolescents. The effects on inhibitory control and cognitive flexibility are considered as small effect sizes, while the effects on working memory are considered as moderate effect size. Limited by the quantity and quality of the included studies, the above conclusions need to be verified with more high-quality studies.

## Introduction

A sedentary lifestyle and physical inactivity (insufficient exercises time) are prevalent among children and adolescents (Sisson et al., 2009; Qi et al., 2019) and are negatively linked with their physical and psycho-cognitive health (Tremblay et al., 2011; Flashner et al., 2019). Specifically, these unhealthy lifestyle behaviors result in obesity (Rey-Lopez, 2008), uncoordinated movements (Ferguson et al., 2014; Flashner et al., 2019; Kong et al., 2019; Riquelme et al., 2019), negative emotions (depression, anxiety, suicide attempts) (Berardelli et al., 2018; Thivel et al., 2018; Padulo et al., 2019) and a severe deficit of cognitive functions (Torrens-Burton et al., 2017; Koolhaas et al., 2019; Loprinzi et al., 2019). Among the components of cognitive functioning, inhibitory control, working memory, and cognitive flexibility play a critical role in the development of school-age children and their educational achievement or academic performance (Best, 2010; Willoughby et al., 2012).

Diamond proposed a three-factor model of executive functions and stated that inhibitory control, working memory, and cognitive flexibility are the three core executive functions (EF); the three aforementioned cognitive abilities work together to influence higher-order executive functions such as reasoning, planning, and problem solving (Diamond, 2013). However, there is little longitudinal experimental evidence in the field of sports and health promotion to prove that physical activity is relevant to high-level executive functions such as decision-making and reasoning in children and adolescents. In contrast, the more such evidence appears in the context of techniques and tactics in high-level athletes (Taddei et al., 2012; Bjoern et al., 2018; Beavan et al., 2020). Therefore, this review only considers the three main executive functions of inhibitory control, working memory, and cognitive flexibility. Firstly, inhibitory control (self-control) refers to the ability to suppress irrelevant reactions, allowing children to control automatic or impulsive behaviors while performing minimal automatic reactions (Pindus et al., 2019); secondly, working memory (refreshing tasks) allows children to register information in the brain and then to perform cognitive operations on it (Keita et al., 2011); thirdly, cognitive flexibility (tasks of conversion) refers to the ability of a child to move flexibly to a new situation or another state (Masley et al., 2009). When EF are impaired, children generally show abnormalities in social functions, emotions, and cognition (Goodall et al., 2018; Wang et al., 2019), often accompanied by learning difficulties, conduct disorders, and maladaptive phenomena (Rocha et al., 2019). Thus, such cognitive abilities in the growth and development stage are undoubtedly crucial for children and adolescents.

Emerging evidence indicates that physical activity and exercise can influence executive functions such as inhibitory control, working memory and cognitive flexibility both acutely and chronically (Rathore and Lom, 2017; De Greeff et al., 2018; Vazou et al., 2019). Furthermore, chronic exercises as a part of healthy lifestyle behaviors are widely recognized to be associated with improved EF across different age groups (Hillman et al., 2011; Li et al., 2017), while the expanding topic on acute exercises (referring to a single bout of exercises taking 10–60 min) indicates their potential to improve these cognitive outcomes (Byun et al., 2014). However, to date, very few reviews have systematically and simultaneously evaluated the effects of both exercise types on the three key EF components in children and adolescents; the reviews on this topic either focused on healthy adults and older adults (Ludyga et al., 2016; McSween et al., 2019; Chen et al., 2020) or chronic exercises alone in the same age group (Ludyga et al., 2016; Xue et al., 2019), or presented unclear information on a selected outcome (reaction time, accuracy, or derived scores) (Li et al., 2017; De Greeff et al., 2018; Sember et al., 2020). Meanwhile, previous studies have shown that physical activity is more sensitive to reaction time than the three key cognitive components (Ellemberg and St-Louis-Deschênes, 2010; Zhu, 2015). Against this background, the primary purpose of this review was to comprehensively analyze the effects of acute and chronic exercises on the EF of children and adolescents and to further explore the effects of acute and chronic exercises on the three different tasks of inhibitory control, working memory, and cognitive flexibility. In response to these differences in the literature, this review highlighted the relationship between exercises and EF, regulated by factors such as exercise type, exercise intensity, exercise duration, individual factors, and subcomponents of executive function.

## Method

Our research follows the requirements of the international meta-analysis writing guidelines (the PRISMA statement for reporting systematic reviews and meta-analyses of studies that evaluate health-care interventions: explanation and elaboration) for selecting and utilizing research methods (Shamseer et al., 2015).

### Retrieval Strategy

The databases PubMed, Web of Science, Scopus, The Cochrane Library, China National Knowledge Infrastructure (CNKI), and Wan Fang were searched from January 1 2009 to December 31 2019. Two reviewers independently searched articles published in Chinese and English, supplemented by a manual search, and retrospectively included references if necessary. The following two sets of search terms were used: “physical activity” or “exercises” or “physical fitness” or “physical endurance” or “motor activity” or “physical education” or “sport” or “basketball” or “football” or “running” or “cycling” or “jumping” or “dancing” or “tai chi” or “yoga” or “aerobic” and “executive function” or “inhibition” or “inhibiting ability” or “self-control” or “working memory” or “updating” or “refreshing” or “cognitive flexibility” or “task-switching” or “shifting” and “child” or “student” or “toddler” or “preschooler” or “adolescents.” If an article was incomplete or unavailable, we contacted the corresponding author by email to obtain detailed information. For literature tracing, based on the retrieved literatures or related references listed in the review, we used Baidu Scholar and Google Scholar to search for them retrospectively.

### Inclusion Criteria and Exclusion Criteria

Two reviewers independently screened the articles. When there was a disagreement between the two reviewers, a third reviewer evaluated the original study to reach a consensus. Any potentially relevant research needed to meet the following inclusion criteria: (1) children and adolescent participants aged 5–18 years with right-hand dominance, corrected or normal vision and a healthy body were deemed eligible; (2) the exercise group was the primary intervention measure (e.g., aerobic-based, motor skill-based, combining aerobic, muscular activity, yoga, basketball), compared with different types of control groups (i.e., no-intervention control group, waiting list, and routine care) and all the intervention measures were motor skill-based or aerobic-based and clearly defined in terms of the exercise protocol; (3) preliminary studies were randomized controlled trials, and the randomization was done either on an individual or on a group (e.g., classroom) basis; (4) outcome indicators included test data on executive function (working memory, inhibition and cognitive flexibility), with a minimum of one outcome with quantitative data for calculating the pooled effect size. Conditions for exclusion from the study included: (1) ambiguous explanations of exercises interventions; (2) irrelevant outcomes; and (3) studies for which the full text could not be obtained.

### Data Extraction

Two reviewers independently extracted data according to a predefined protocol. If there were any differences or inconsistencies, they would discuss the study with a third reviewer. They gathered the following information: (1) literature information, including author name, year of publication and country; (2) sample size (male sample); (3) socioeconomic status; (4) age of subjects, mainly used to divide the type of population; (5) intensity of exercise intervention, duration of intervention, time of intervention, frequency of intervention; (6) intervention program; (7) measurements, mainly including the three dimensions of working memory, inhibition and cognitive flexibility; and (8) adverse events and follow-up.

### The Methodological Quality of the Included Studies

Similar to previous studies (Zou et al., 2018, 2019), the Physiotherapy Evidence Database Scale (PEDro) was used to assess the risk of bias (Macedo et al., 2010). The assessment tool includes 10 items, as follows: eligibility criteria, randomization, concealed allocation, similar baseline, assessor blindness, subject blindness, point estimation, comparison between groups, a retention rate of 85% or above and completeness of measurement results. Notably, the use of a blinded instructor was unrealistic during exercise interventions, and so this item was not considered. A higher total score (0–10 points) represents a better methodological quality, where a PEDro score ≥6 is categorized as the high-quality group while a PEDro score <6 is categorized into the low-quality group (Maher et al., 2003). The methodological quality of the included studies was independently assessed by two panelists using PEDro, and any differences were resolved by a third reviewer.

### Statistical Analysis

Stata 12.0 (Stata Corp, College Station, TX) was used as data processing software (Press, 2009). We used the standardized mean difference (SMD) with a 95% confidence interval (CI) to analyze the combined effect size. According to the Cochrane systematic review manual, if a study included more than one control group, the sample size of the exercise intervention should be equally assigned during pair comparison in order to avoid analysis unit errors (Handoll et al., 2002). If statistical heterogeneity was found across studies (*I*^2^ ≥ 50%, *p* < 0.10), we applied the random-effects model—otherwise, the fixed-effect model was applied—and we used the Hedges' g method to reflect the magnitude of exercise intervention (Liu et al., 2019). According to the criteria for evaluating effect volumes, a small effect was between ≥0.2 and <0.5, a medium effect was between ≥0.5 and <0.8, and a large effect was ≥0.8 (Hanley et al., 2003). The heterogeneity of the included studies was determined with the *p*-value (threshold point of 0.1) and *I*^2^ statistics (25, 50, and 75%, representing small, medium, and large heterogeneity, respectively) (Liu et al., 2019).

Given that overall effect sizes may be influenced by heterogeneity factors (age, study quality, motor skill type, composite type, intervention duration, intervention frequency, and intervention time), several regression analyses were separately performed. Additionally, subgroup analyses were also performed for age, study quality, motor-skill type, composite type, and intervention duration, frequency, and time: (1) prepubertal children (5–12 years) vs. adolescents (12–18 years) (Cardoso, 2007); (2) PEDro scores of >6 points (high-quality studies) vs. PEDro scores of <6 (low-quality studies) (Maher et al., 2003); (3) open-skilled exercises (different types of motor skills that respond to individual requirements in a dynamically changing or unpredictable external environment, where physical education, basketball, and ping-pong belong to open exercises) vs. closed-skill exercises (movements with relatively stable sports environments, such as yoga and running) (Liu et al., 2019); (4) sole-mode training (the use of a single skill) vs. multimodal training (the use of a variety of skills including yoga and running, aerobic exercises); (5) 12 weeks was used as a cutoff for chronic exercise intervention; (6) intervention time referred to the timing of intervention, where a threshold of 30 min for chronic exercise was recommended; and (7) three times a week was used as a threshold for chronic exercise intervention frequency. The selection of these moderators was principally inspired by McMinn and Rathore (Mcminn, 2012; Rathore and Lom, 2017).

### Evidence Certainty Assessment

The Grading Recommendations to Assess Development and Evaluation system (GRADE) is an evidence evaluation system and is one of the international standards for evidence quality and the classification of recommendation strength (Zhang et al., 2019). We evaluated the quality of the evidence for each outcome using the GRADE classification with four possible levels: I (high), where the real effect is similar to a credible estimate; II (moderate), where the true effect is closest to the estimated effect; III (low), where the actual effect may be significantly different from the estimated effect; and IV (very low), where the actual effect is likely to be significantly different from the estimated effect. Five factors can cause the quality of the evidence to decrease: (1) risk of bias; (2) imprecision; (3) inconsistency; (4) indirectness; and (5) publication bias.

## Results

### Literature Search Results

**Figure 1:**
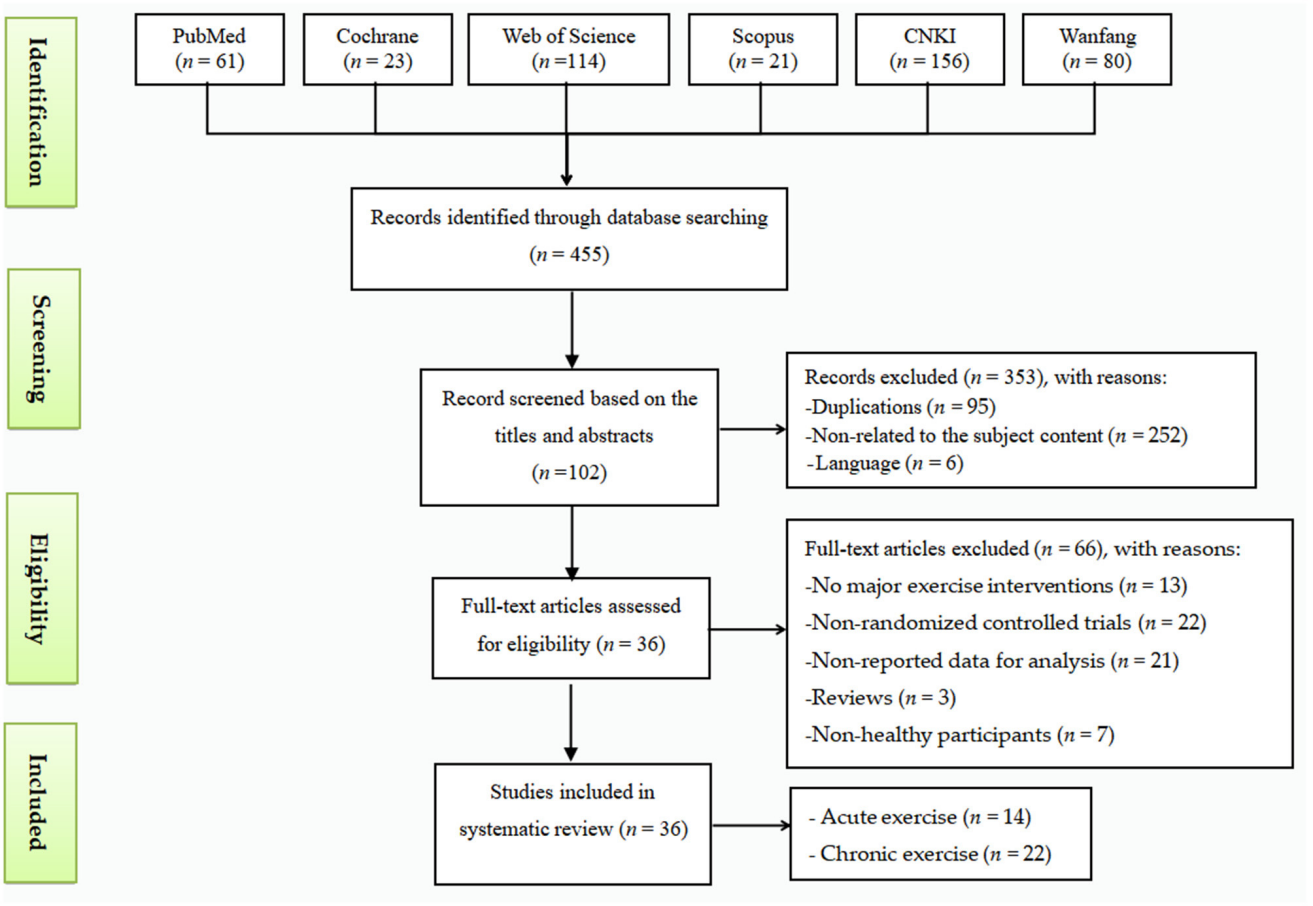
Flow of the study selection method.

The latest review of electronic searches (as of December 2019) retrieved a total of 455 articles. During the preliminary screening, we excluded 353 studies based on their title and abstract for reasons including duplications (*n* = 95), language (*n* = 6), or not being related to the subject content (*n* = 252). Further screening was performed by reading the full text, and 66 records were excluded because of non-randomized controlled trials (*n* = 22), no reported data for analysis (*n* = 21), review (*n* = 3), no major exercise interventions (*n* = 13), or non-healthy participants (*n* =7). Finally, the meta-analysis included 36 articles: 14 for acute exercises and 22 for chronic exercises (Figure 1).

### Eligible Research Features

The 36 articles included were randomized controlled trials (14 acute exercises, 22 chronic exercises), including 4,577 healthy participants (1,308 participants of acute exercises), of which 2,227 were in the experimental group (670 participants of acute exercises) and 2,350 were in the control group (635 participants of acute exercises) (Tables 1, 2). In acute exercises and chronic exercises, the ages of the included students ranged from 5 to 18 years old, while three of the studied articles did not specify age (Chaddock-Heyman et al., 2013; Weng et al., 2014; Budde et al., 2016). In terms of interventions, 12 studies used acute exercises—short-term, medium-intensity aerobic exercises, such as jogging or power cycling, with an exercise time from 10 to 40 min—and 20 studies used chronic exercise interventions with a duration from 8 to 20 weeks, 2–5 times a week, and with 30–90 min per session. Furthermore, each study consisted of at least one dimension of the outcome indicators of inhibitory control (10 acute exercises, 15 chronic exercises), working memory (nine acute exercises, 13 chronic exercises), and cognitive flexibility (three acute exercises, eight chronic exercises) in three dimensions. In addition, only three articles in the included studies reported follow-up status (Fisher et al., 2011; Telles et al., 2013; Tarp et al., 2016), and no adverse events occurred.

**Table 1:** Characteristics of the studies included in the meta-analysis (acute exercises).

**References**	**Location (Language)**	**Participant characteristics**			**Intervention program**	**Intervention characteristics**		**Outcome measured**	**Adverse event;** ** Follow-up**
		***N* (male)**	**SES**	**Mean age or Age range**		**Time** ** (min)**	**Motion intensity**		
Benzing et al. (2016)	Bern, Switzerland (English)	65 (34)	NR	14.51 ± 1.08	EG: Run + jump + resistance exercises (aerobic-based) CG: Usual care	15	60%−70%	(Inhibition) (Fluency) (Cognitive flexibility)	No No
Pate (2015)	Carolina, USA (English)	96 (37)	NR	10.70 ± 0.60	EG: Better ideas through exercises (aerobic-based) CG: Usual care	10–20	NR	(Digit Recall)	No No
Gothe et al. (2013)	Urbana, USA (English)	40 (20)	NR	9.50 ± 0.50	EG: Yoga (motor skill-based) CG: Usual care	20	60%−70%	(Stroop Test)	No No
Chen et al. (2015)	Yangzhou, China (Chinese)	130 (53)	NR	9.40 ± 0.30	EG: Basketball (motor skill-based) CG: Usual care	15–30	65%	(Flanker task) (1-back) (More-odd shifting)	No No
Ellemberg and St-Louis-Deschênes (2010)	Montreal, Canada (English)	72 (38)	NR	7.75 ± 0.65	EG: Basketball (motor skill-based) CG: Usual care	40	63%	(Flanker task)	No No
Jager et al. (2015)	Bern, Switzerland (English)	219 (112)	6.90 (1.56)	11.35 ± 0.65	EG: Run + bicycle (combining aerobic and muscular activity) CG: Usual care	30	70%	Wisconsin Card Sorting Test	No No
Kubesch et al. (2009)	Ulm, Germany (English)	81 (NR)	NR	13–14	EG: Run + resistance exercises (aerobic-based) CG: Usual care	30	NR	(Flanker task) (1-back)	No No
Chun et al. (2015)	Taiwan, China (English)	22 (9)	NR	15.42 ± 1.47	EG: Bicycle (aerobic-based) CG: Usual care	30	65%	Wisconsin Card Sorting Test	No No
Budde et al. (2010)	Berlin, Germany (English)	60 (0)	NR	14.37 ± 0.53	EG: Run (aerobic-based) CG: Usual care	12	50%-85%	Digit Span task	No No
Yan et al. (2014)	Yangzhou, China (Chinese)	244 (113)	NR	9.50 ± 0.30	EG: Run (aerobic-based) CG: Usual care	30	60%−69%	(Go/no-go) (1-back) (More-odd shifting)	No No
Cooper et al. (2018)	Nottingham, UK (English)	41 (NR)	NR	12.30 ± 0. 71	EG: Basketball (motor skill-based) CG: Usual care	60	60%−70%	(Stroop test) (Sternberg paradigm)	No No
Park and Etnier (2019)	Daejeon, Korea (English)	22 (NR)	NR	15.90 ± 0. 29	EG: Better ideas through exercises (aerobic-based) CG: Usual care	20	60%−70%	(Stroop Test)	No No
Egger et al. (2018)	Bern, Switzerland (English)	216 (110)	NR	7.94 ± 0. 44	EG: Better ideas through exercises (aerobic-based) CG: Usual care	20	NR	(Stroop Test)	No No
Vera et al. (2018)	Netherlands, Amsterdam (English)	38 (12)	NR	12.30 ± 0.60	EG: Bicycle (aerobic-based) CG: Usual care	20-30	40%−60%	(N-back)	No No

*NR = not reported; EG = experimental group; CG = control group; SES = socioeconomic status; ① represents inhibitory control; ② represents working memory; ③ represents cognitive flexibility*.

**Table 2:** Characteristics of the studies included in the meta-analysis (chronic exercises).

**Reference**	**Location (language)**	**Participant characteristics**			**Intervention program**	**Intervention characteristics**			**Outcome measured**	**Adverse event;follow-up**
		***N* (male)**	**SES**	**Mean age orage range**		**Time(min)**	**Frequency (weekly)**	**Duration(week)**		
Chen et al. (2016)	Yangzhou, China (Chinese)	40 (20)	NR	11.36 ± 0.57	EG: Aerobic dance CG: Usual care	40	3	8	(Flanker task) (1-back) (More-odd shifting)	No No
De Greeff et al. (2016)	Groninge, Netherlands (English)	499 (216)	NR	8.20 ± 0.70	EG: Aerobic exercises CG: Usual care	20–30	3	22	Wisconsin Card Sorting Test	No No
Kval et al. (2017)	Stavanger, Norway (English)	429 (NR)	NR	10–11	EG: Jump rope + running + strength training (combining aerobic and muscular activity) CG: Usual care	45	2	10	(Stroop Test)	No No
Jiang (2015)	Beijing, China (Chinese)	61 (25)	NR	5.56 ± 0.35	EG: Football (motor skill-based) CG: Usual care	35	2	8	(Flexible-Item Selection)	No No
Xin (2012)	Shandong, China (Chinese)	40 (20)	NR	9.10 ± 0.32	EG: Tennis (motor skill-based) CG: Usual care	40	5	16	(Flanker task); (1-back) (More-odd shifting)	No No
Budde et al. (2016)	Hamburg, Germany (English)	71 (32)	NR	9.35± 0.60	EG: Run + jump + resistance exercises (aerobic-based) CG: Usual care	45	3	10	(Letter Digit Span)	No No
Purohit and Pradhan (2016)	Bengaluru, India (English)	72 (30)	NR	12.69 ± 1.35	EG: Yoga (motor skill-based) CG: Usual care	90	4	12	(Stroop Test) (More-odd shifting)	No No
Wang (2017)	Beijing, China (Chinese)	30 (14)	NR	5–6	EG: Tennis (motor skill-based) CG: Usual care	60	2	8	(Flanker task) (1-back)	No No
Yin et al. (2018)	Beijing, China (Chinese)	326 (165)	NR	7–9	EG: Run (aerobic-based) EG: Taijiquan (aerobic-based) CG: Usual care	30	3–5	20	(Flanker task) (1-back) (More-odd shifting)	No No
Chaddock-Heyman et al. (2013)	Urbana, USA (English)	26 (11)	2.32 (1.09)	NR	EG: Bicycle (aerobic-based) CG: Usual care	76.8	5	22	(1-back) (More-odd shifting)	No No
Stroth et al. (2009)	Ulm, Germany (English)	35 (NR)	NR	14.20 ± 0.50	EG: Aerobic exercises CG: Usual care	40	3	12	(1-back)	No No
Lina (2017)	Yangzhou, China (Chinese)	17 (9)	NR	11.37 ± 1.53	EG: Run (aerobic-based) CG: Usual care	30	4	11	(0-back)	No No
Yan Jun and Chen (2013)	Yangzhou, China (Chinese)	87 (42)	NR	9.50 ± 0.30	EG: Aerobic dance CG: Usual care	30	3	12	(Flanker task) (1-back) (More-odd shifting)	No No
Keita et al. (2011)	Illinois, USA (English)	36 (19)	NR	7–9	EG: Medicine balls + resistance exercises (combining aerobic and muscular activity) CG: Usual care	70	5	24	(Reaction time)	No No
Hillman et al. (2014)	Illinois, USA (English)	221 (NR)	NR	8–9	EG: Yoga + run (aerobic-based) CG: Usual care	70	5	24	(Flanker task) (Cognitive flexibility)	No No
Telles et al. (2013)	Uttarakhand, India (English)	98 (60)	NR	10.50 ± 1.30	EG: Yoga (aerobic-based) CG: Usual care	45	5	12	(Stroop Test)	No Yes
Fisher et al. (2011)	Glasgow, UK (English)	64 (29)	7(1)	6.10± 0.30	EG: Run (aerobic-based) CG: Usual care	120		10	(Reaction time)	No Yes
Tarp et al. (2016)	Rotterdam, Netherlands (English)	698 (309)	NR	12.90± 0.60	EG: Whole-body movement games CG: Usual care	60	4	12	(Reaction time)	No Yes
Ludyga et al. (2017)	Basel, Switzerland (English)	36 (18)	NR	12-15	EG: Medicine balls + relay games (combining aerobic and coordinative exercises)	5-10	5	8	(Stroop Test)	No Yes
Nie (2019)	Nanjing, China (Chinese)	40 (19)	NR	13.81± 0.30	EG: Wuqinxi (aerobic-based) CG: Usual care	45	3	8	(Flanker task) (1-back) (More-odd shifting)	No Yes
Egger et al. (2019)	Bern, Switzerland (English)	142 (70)	NR	7.91± 0.40	EG: Better ideas through exercises (aerobic-based) CG: Usual care	20	5	20	(Flanker task)	No No
Vera et al. (2019)	Netherlands, Amsterdam (English)	201 (108)	NR	10.90± 0.70	EG: Dance (aerobic-based) CG: Usual care	10	5	9	(Stroop Test)	No No

*NR = not reported; EG = experimental group; CG = control group; SES = socioeconomic status; ① represents inhibitory control; ② represents working memory; ③ represents cognitive flexibility*.

### Methodological Quality Evaluation

The methodological quality of the included studies is presented in Table 3. The mean scores for acute and chronic exercise types were 7 and 6.77, respectively, indicating a high degree of credibility. All 36 studies were randomized controlled trials, five articles described the method for hiding random allocation (Kubesch et al., 2009; Ellemberg and St-Louis-Deschênes, 2010; Telles et al., 2013; Hillman et al., 2014; Budde et al., 2016), and the rest only mentioned random allocation. Eight studies adopted the blind-reviewer method (Fisher et al., 2011; Telles et al., 2013; Hillman et al., 2014; Chun et al., 2015; Jager et al., 2015; Budde et al., 2016; Chen et al., 2016; Yin et al., 2018), and five articles used the blind-examiner method (Fisher et al., 2011; Telles et al., 2013; Yan et al., 2014; Jager et al., 2015; Chen et al., 2016); the rest were implemented without a blind method. There were 11 articles that did not describe the source of their examination.

**Table 3:** Physiotherapy Evidence Database Scale (PEDro) of the included randomized controlled trials (acute exercises and chronic exercises).

**Author [Reference]**	**Item 1**	**Item 2**	**Item 3**	**Item 4**	**Item 5**	**Item 6**	**Item 7**	**Item 8**	**Item 9**	**Item 10**	**Score**
Benzing et al. (2016)	1	1	0	1	0	0	1	1	1	1	7
Pate (2015)	1	1	0	0	0	0	1	1	1	1	6
Gothe et al. (2013)	1	1	0	0	0	0	1	1	1	1	6
Chen et al. (2015)	1	1	0	1	0	0	1	1	1	1	7
Ellemberg and St-Louis-Deschênes (2010)	0	1	1	1	0	0	1	1	1	1	7
Jager et al. (2015)	1	1	0	0	1	1	1	1	1	1	8
Kubesch et al. (2009)	1	1	1	1	0	0	1	1	1	1	8
Chun et al. (2015)	1	1	0	1	1	0	1	1	1	1	8
Budde et al. (2010)	1	1	0	1	0	0	1	1	1	1	6
Yan et al. (2014)	0	1	0	1	0	1	1	1	1	1	7
Cooper et al. (2018)	1	1	0	1	0	0	1	1	1	1	7
Park and Etnier (2019)	1	1	0	1	0	0	1	1	1	1	7
Egger et al. (2018) Vera et al. (2018)	1	1	0	1	0	0	1	1	1	1	7
Vera et al. (2018)	1	1	0	1	0	0	1	1	1	1	7
***Mean Score (acute exercises)***											**7.00**
Chen et al. (2016)	1	1	0	1	1	1	1	1	1	1	9
De Greeff et al. (2016)	0	1	0	1	0	0	1	1	1	1	6
Kval et al. (2017)	1	1	0	1	0	0	1	1	1	1	7
Jiang (2015)	1	1	0	0	0	0	1	1	1	1	6
Xin (2012)	0	1	0	1	0	0	1	1	1	1	6
Budde et al. (2016)	1	1	1	1	0	0	1	1	1	1	8
Purohit and Pradhan (2016)	0	1	0	0	0	0	1	1	1	1	5
Wang (2017)	0	1	0	1	0	0	1	1	1	1	6
Yin et al. (2018)	1	1	0	1	1	0	1	1	1	1	8
Chaddock-Heyman et al. (2013)	1	1	0	1	0	0	1	1	1	1	7
Stroth et al. (2009)	1	1	0	0	0	0	1	1	1	0	5
Lina (2017)	0	1	0	1	0	0	1	1	1	1	6
Yan Jun and Chen (2013)	0	1	0	0	0	0	1	1	1	1	5
Keita et al. (2011)	0	1	0	1	0	0	1	1	1	1	6
Hillman et al. (2014)	1	1	1	1	1	0	1	1	1	1	9
Telles et al. (2013)	1	1	1	1	1	1	1	1	1	1	10
Fisher et al. (2011)	0	1	0	1	1	1	0	1	1	1	7
Tarp et al. (2016)	0	1	0	1	0	0	0	1	1	1	5
Ludyga et al. (2018)	1	1	0	1	0	0	1	1	1	1	7
Nie (2019)	1	1	0	1	0	0	1	1	1	1	7
Egger et al. (2019)	1	1	0	1	0	0	1	1	1	1	7
Vera et al. (2018)	1	1	0	1	0	0	1	1	1	1	7
***Mean Score (chronic exercises)***											**6.77**

*Item 1 = eligibility criteria; Item 2 = randomization; Item 3 = concealed allocation; Item 4 = similar baseline; Item 5 = assessor blindness; Item 6 = subject blindness; Item 7 = a retention rate of 85% or above; Item 8 = comparison between groups; Item 9 = point measure and measures of variability; Item 10 = completeness of measurement results (“1” means that the corresponding item was explicitly described and present in details; “0” means that the corresponding item was absent, inadequately described, or unclear)*.

## Meta-Analysis Results

**Figure 2:**
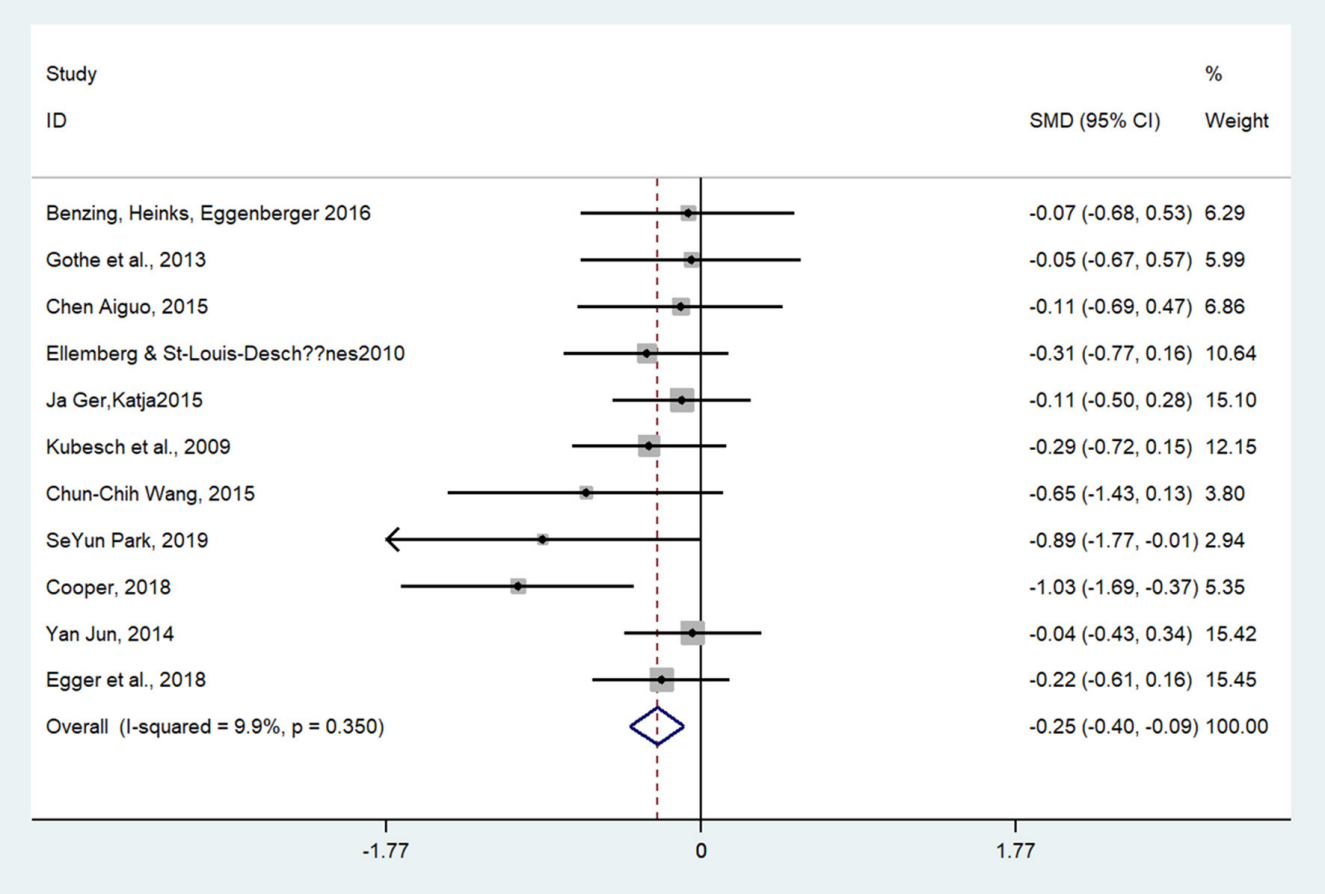
The effect of acute exercises on inhibitory control (SMD = standardized mean difference; CI = confidence interval).

**Figure 3:**
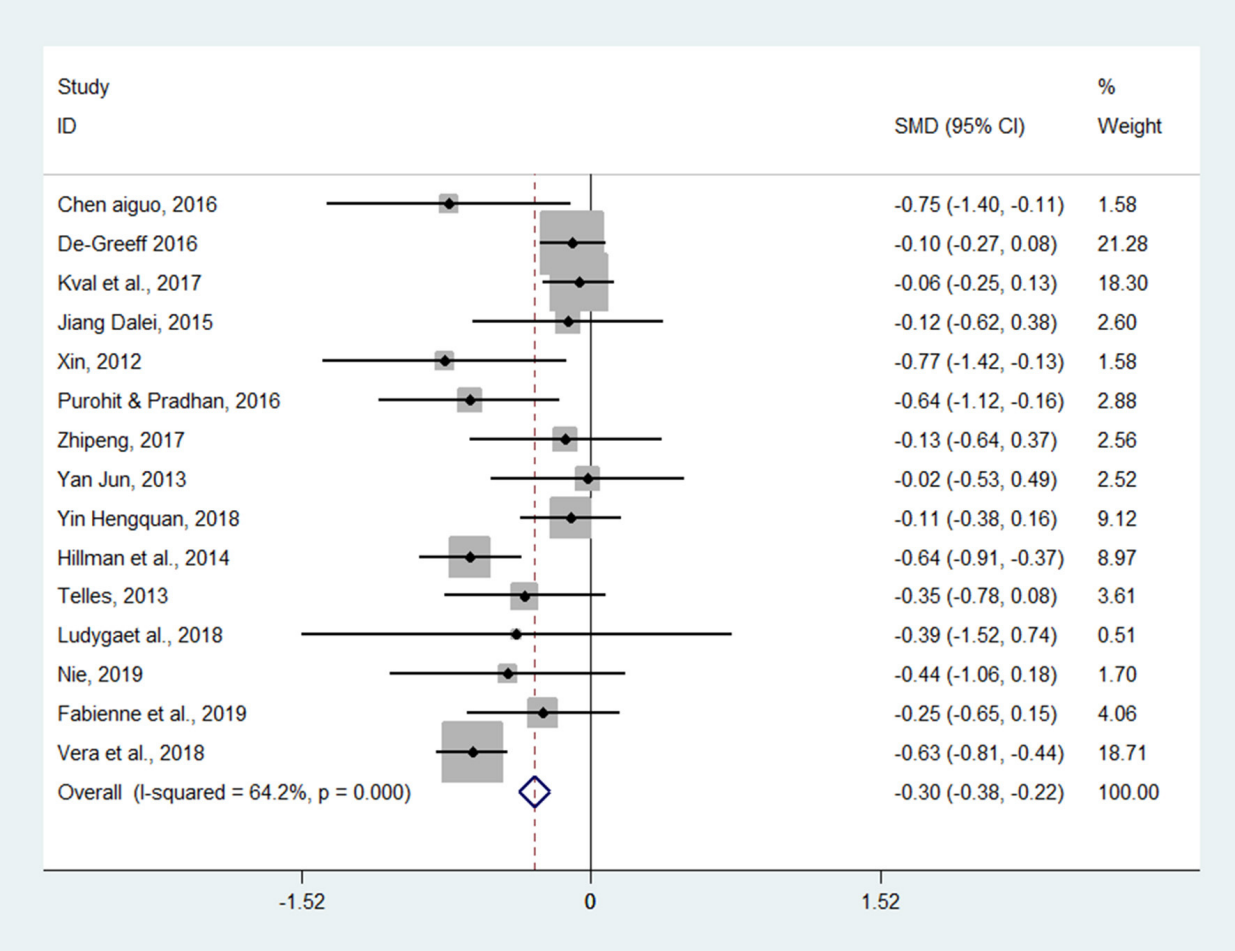
The effect of chronic exercises on inhibitory control (SMD = standardized mean difference; CI = confidence interval).

Twenty-three studies examined the effect of exercises on inhibitory control (as measured by the Stroop, Go/no-go, or Flanker tasks). The aggregated result showed a significant benefit in favor of acute exercises on the inhibitory control of children and adolescents (SMD = −0.25; 95% CI −0.40 to −0.09, *I*^2^ = 9.9%, *p* = 0.002) (Figure 2). The aggregated result showed that chronic exercises can significantly shorten response times (SMD = −0.30; 95% CI −0.38 to −0.22, *I*^2^ = 64.2%, *p* <0.001) (Figure 3). The SMDs of acute and chronic exercises were considered as small ESs.

**Figure 4:**
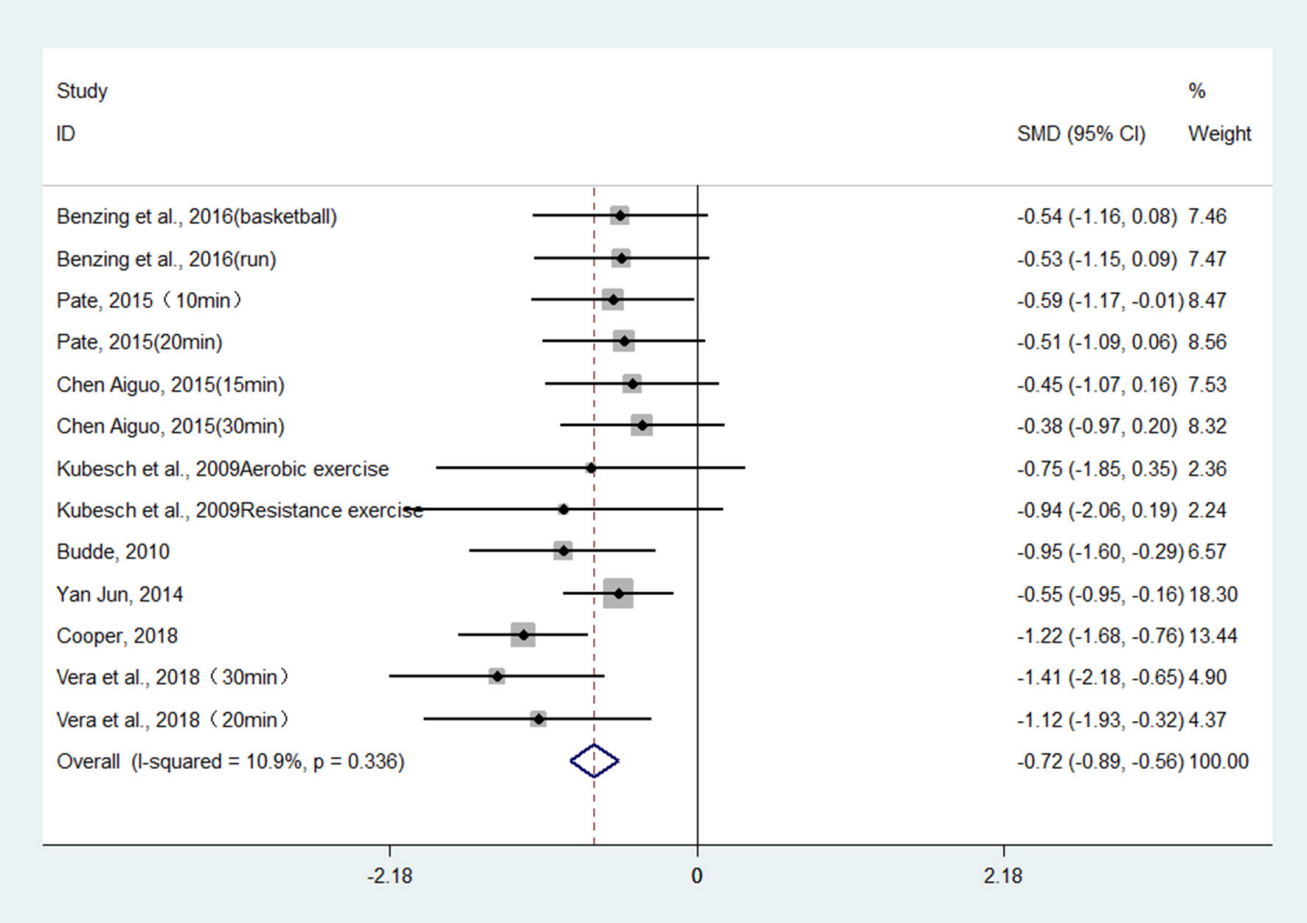
The effect of acute exercises on working memory (SMD = standardized mean difference; CI = confidence interval).

**Figure 5:**
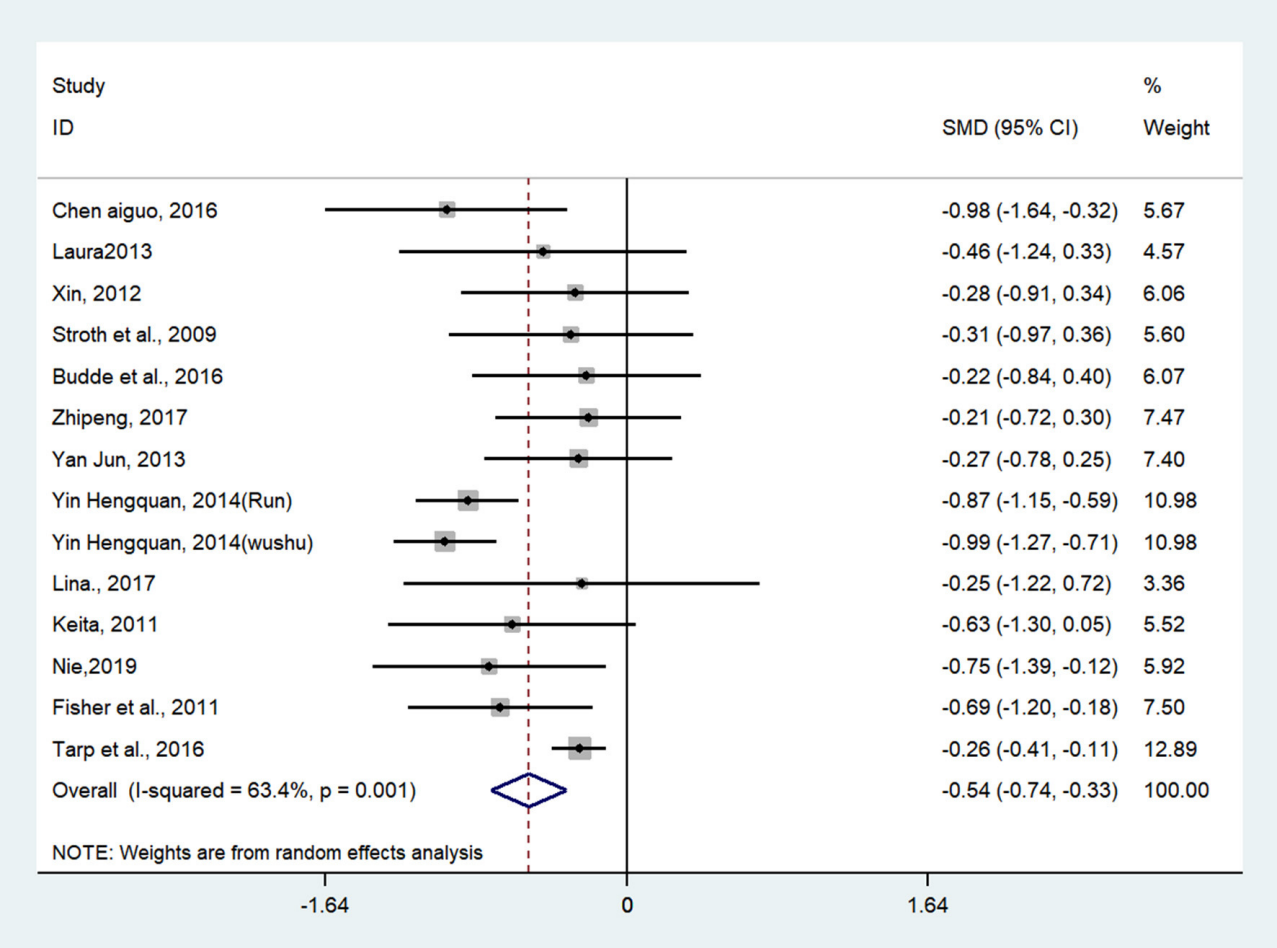
The effect of chronic exercises on working memory (SMD = standardized mean difference; CI = confidence interval).

Twenty-two studies (27 pairwise comparisons) examined the effect of exercises on working memory (as measured by digit span backward, Tower of London, and N-back tasks). A higher negative value of the mean change score for the reaction time indicated less time being required for working memory. The aggregated result showed a significant benefit in favor of acute exercises on working memory (SMD = −0.72; 95% CI −0.89 to −0.56, *I*^2^ = 10.9%, *p* < 0.001) (Figure 4). Chronic exercises were shown to shorten the response time effectively (SMD = −0.54; 95% CI −0.74 to −0.33, *I*^2^ = 63.4%, *p* < 0.001) (Figure 5). The SMDs of acute and chronic exercises were considered as moderate ESs.

**Figure 6:**
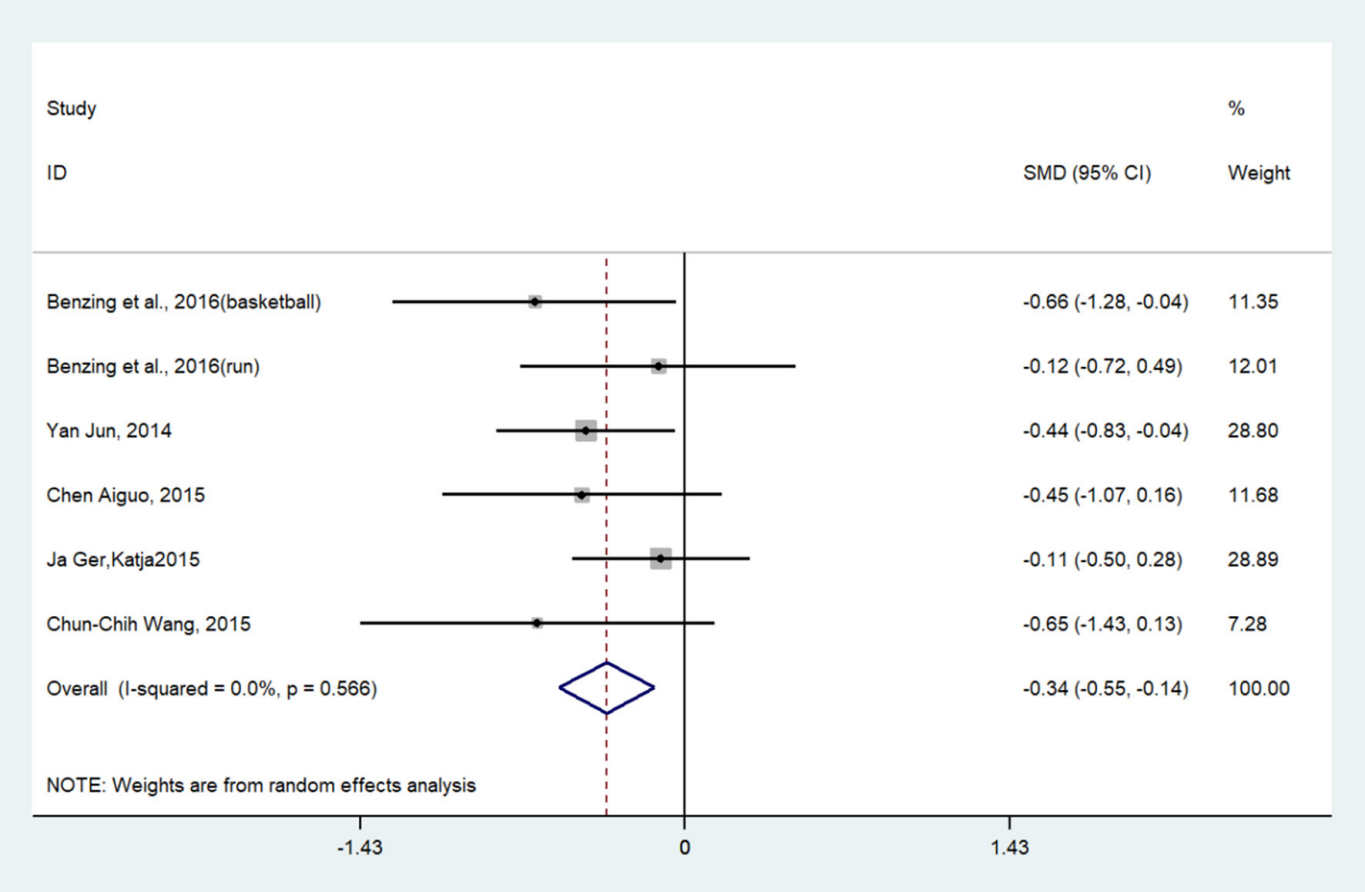
The effect of acute exercises on cognitive flexibility (SMD = standardized mean difference; CI = confidence interval).

**Figure 7:**
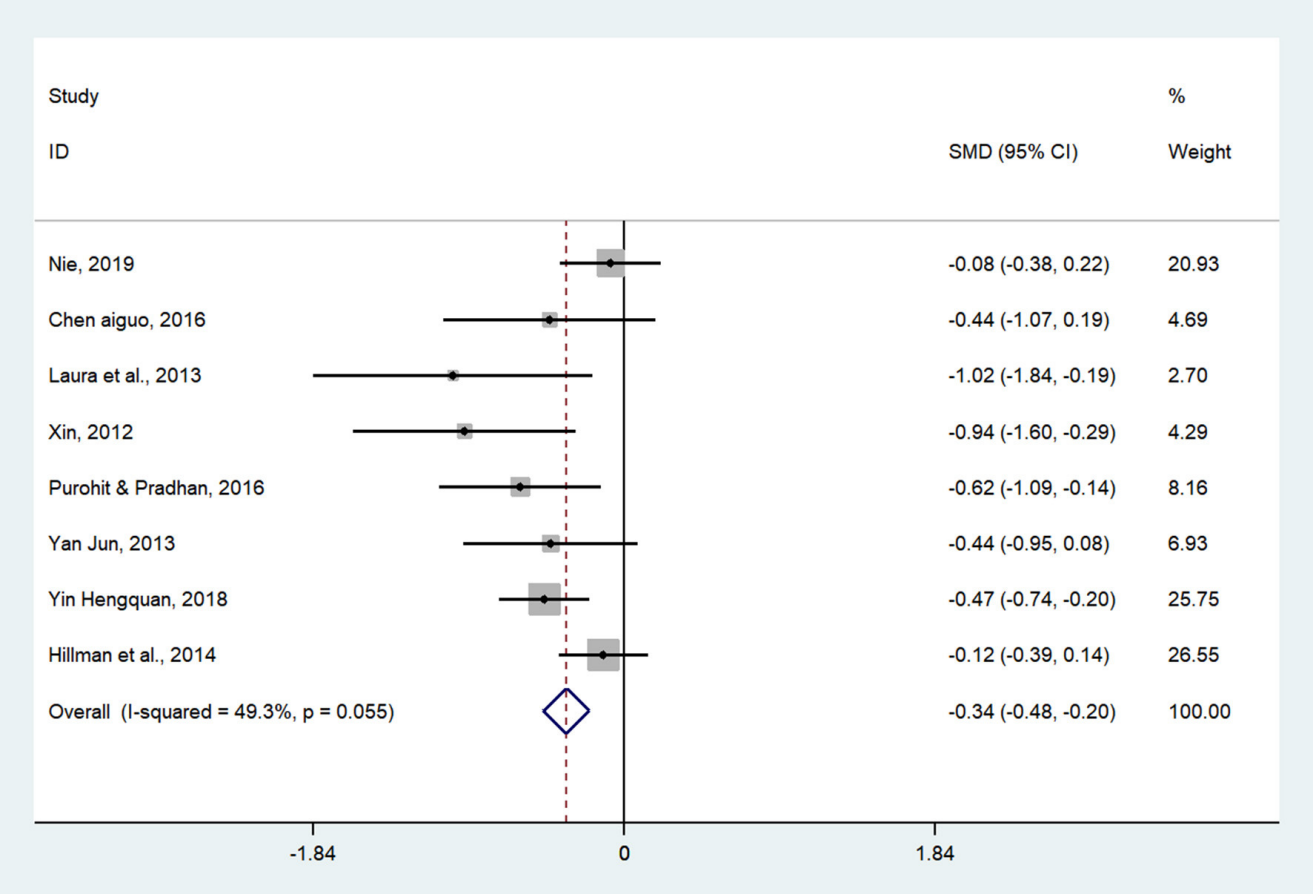
The effect of chronic exercises on cognitive flexibility (SMD = standardized mean difference; CI = confidence interval).

Thirteen studies (14 pairwise comparisons) examined the effect of exercises on cognitive flexibility (as measured by more-odd shifting and the Wisconsin card sorting test). A higher negative value of the mean change score for the reaction time indicated less time being required for cognitive flexibility. The aggregated result showed a significant benefit in favor of acute exercises on cognitive flexibility (SMD = −0.34; 95% CI −0.55 to −0.14, *I*^2^ = 0%, *p* < 0.005) (Figure 6). Similarly, chronic exercises also had a significant effect on cognitive flexibility (SMD = −0.34, 95 % CI −0.48 to −0.20, *I*^2^= 49.3%, *p* < 0.001) (Figure 7). The SMDs of acute and chronic exercises were considered as small ESs.

### GRADE Quality Evaluation

Based on the criteria of GRADE, the assessment of the certainty of the evidence regarding the significant impact of exercises on the subsets (inhibitory control, working memory, and cognitive flexibility) of executive functions in children and adolescents was separately evaluated (Table 4). Specifically, acute exercises exhibited medium-quality evidence for the working memory, inhibitory control, and cognitive flexibility of children and adolescents in their executive functions, whereas chronic exercises showed medium-quality evidence on inhibitory control and low-quality evidence on working memory and cognitive flexibility.

**Table 4:** Grading Recommendations to Assess Development and Evaluation (GRADE) assessment of the evidence of certainty for exercise effects.

**Outcomes**		**Presence of downgrading item of GRADE**					**Level of certainty of evidence**
		**Risk of bias**	**Inconsistency**	**Indirectness**	**Imprecision**	**Publication bias**	
Chronic exercises	Inhibitory control	Yes	No	No	No	No	II (moderate) (1)
	Working memory	Yes	No	No	Yes	No	III (low) (1) (4)
	Cognitive flexibility	Yes	No	No	Yes	No	III (low) (1) (4)
Acute exercises	Inhibitory control	Yes	No	No	No	No	II (moderate) (1)
	Working memory	Yes	No	No	No	No	II (moderate) (1)
	Cognitive flexibility	Yes	No	No	No	No	II (moderate) (1)

*(1) Risk of bias: if the risk of bias of the included studies is present in the meta-analysis, e.g., randomization, concealed allocation, or blinding of assessors/subjects; (2) Inconsistency: point estimates are concentrated, confidence intervals can overlap, and the results of the heterogeneity tests are not statistically significant; (3) Indirectness: present if the intervention studied in the meta-analysis is not directly relevant to the outcome; (4) Imprecision: present if the sum of sample sizes of all individual studies included in the meta-analysis is less than 500, and if the effect size's 95% Cl is comparatively large; (5) Publication bias: present if the author only searched the Chinese database, or only one database*.

### Moderator Analysis

For chronic exercises, variables (age, type, study quality, composite, frequency, time, and duration) are likely to be the influencing factors for children and adolescents on their inhibitory control and working memory. Moderator analysis using separate models was employed to examine potential sources of variance. All results are presented in Table 3.

#### Inhibitory Control Moderators

In terms of the composite type of intervention, either multiple exercise interventions or sole exercise interventions were employed in the original studies. There were no statistically significant differences on the ESs between the two types of interventions (Q = 10.25, *p* = 0.001) (Table 5). For chronic exercises, the sole exercise interventions had a significant improvement on inhibitory control (SMD =-0.55, 95% CI −0.83 to −0.25, *p* < 0.001) compared with the multiple exercise interventions (SMD = −0.16, 95% CI −0.27 to −0.06, *p* < 0.001). Furthermore, for intervention classification in the experimental group, open motor skills and closed motor skills were included in our current meta-analysis, showing a statistically significant difference in the ES (Q = 14.49, *p* = 0.001). The results showed a significant effect of open motor skills (SMD = −0.56, 95% CI −0.73 to −0.40) on inhibitory control. In terms of age, there was a statistically significant difference (Q = 12.23, *p* = 0.01). A large and significant reduction in the ES was attributed to children 12–18 years old (SMD = −0.81, 95% CI −1.15 to −0.48, *p* < 0.001) when compared with children 5–12 years old (SMD = −0.19, 95% CI −0.28 to −0.09, *p* < 0.01). Moreover, there were no significant differences in the duration (Q = 0.07, *p* = 0.791), frequency (Q = 1.48, *p* = 0.224), and exercise session time (Q = 3.57, *p* = 0.110). Similarly, the quality of studies did not produce a statistically significant difference between the two levels (Q = 2.39, *p* = 0.124).

**Table 5:** Subgroup analysis of inhibitory control (chronic exercises). SMD: standardized mean difference.

**Subgroup**	***N***	**SMD**	**95% conf. interval**	***I^**2**^***	**Test for between-group heterogeneity**		
					**Q-value**	**df (Q)**	***p*-value**
**Age**							
5–12	11 (1567)	−0.20	−0.28, −0.09	50.8%	12.23	1	0.001
12–18	3 (148)	−0.81	−1.15, −0.48	72.5%			
**Type**							
Open motor skills	9 (859)	−0.56	−0.73, −0.40	60.1%	14.49	1	0.001
Closed motor skills	6 (856)	−0.11	−0.22, 0.00	0.0%			
**Study quality**							
Scores more than 6 (>6)	8 (716)	−0.32	−0.55, −0.20	25.2%	2.39	1	0.124
Scores less than 6 (≤6)	7(998)	−0.18	−0.31, −0.06	84.7%			
**Duration**							
More than 12 weeks	7 (545)	−0.22	−0.36, −0.09	69.6%	0.07	1	0.791
Less than 12 weeks	8(1170)	−0.25	−0.37, −0.12	72.8%			
**Composite type**							
Multiple exercises intervention	7 (713)	−0.16	−0.27, −0.06	64.2%	10.25	1	0.001
Sole exercises intervention	8 (1002)	−0.55	−0.83, −0.25	55.8%			
**Frequency**							
1–3 times/week	5(442)	−0.14	−0.25, −0.05	69.0%	1.48	1	0.224
4–7 times/week	10(1273)	−0.42	−0.58,−0.27	49.8%			
**Exercise session time**							
≤30	5(489)	−0.12	−0.24, 0.05	0.0%	3.57	1	0.110
>30	10(1226)	−0.47	−0.73, −0.21	70.0%			

#### Working Memory Moderators

In terms of intervention classification, a statistically significant difference of the evaluated ES was observed (Q = 20.53, *p* = 0.001) in open motor skills (SMD = −0.72, 95% CI −0.93 to −0.43, *p* < 0.001) and closed motor skills (SMD = −0.31, 95% CI −0.57 to −0.25, *p* < 0.01) (Table 6). In terms of the composite type of intervention, the two types of composite (multiple exercise interventions and sole exercise interventions) did not contribute to statistically significant differences for the ES estimate (Q = 1.47, *p* = 0.257), where both multiple exercise interventions (SMD =-0.58, 95% CI −1.15 to −0.37, *p* < 0.001) and sole exercise interventions (SMD = −0.44, 95% CI −0.83 to −0.25, *p* < 0.001) led to significant improvements in working memory. Notably, study quality produced a significant difference in working memory (Q = 27.89, *p* = 0.001). A study quality score of more than 6 had a small and significant ES (SMD = −0.33, 95% CI −0.55 to −0.21, *p* < 0.01). By contrast, a large and significant ES on working memory was found in favor of studies with a quality score of <6 (SMD = −0.86, 95% CI −1.13 to −0.39, *p* < 0.01). In addition, the factor of age contributed to statistically significant differences for the ES estimate (Q = 18.06, *p* = 0.001); children 5–12 years old had a greater improvement in working memory (SMD = −0.64, 95% CI −0.87 to −0.42, *p* < 0.001) than those 12–18 years old (SMD = −0.30, 95% CI −0.49 to −0.12, *p* < 0.001). Additionally, in terms of duration, frequency, and exercise session time, there was a statistically significant difference for exercise session time (Q = 18.92, *p* = 0.001). A moderate and significant reduction in the ES was attributed to the exercise session time (≤30 min, SMD =-0.82, 95% CI −1.01 to −0.64, *p* < 0.001) when compared with prolonged exercise (>30 min), which contributed to a small ES (SMD =0.35, 95% CI −0.47 to −0.22, *p* < 0.001). By contrast, statistically significant differences of the evaluated ESs were not observed for duration (Q = 0.16, *p* = 0.694) and frequency (Q = 0.10, *p* = 0.953).

**Table 6:** Subgroup analysis of working memory (chronic exercises).

**Subgroup**	***N***	**SMD**	**95% conf. interval**	***I^**2**^***	**Test for between-group heterogeneity**		
					**Q-value**	**df (Q)**	***p*-value**
**Age**							
5–12	9 (638)	−0.64	−0.87, −0.42	47.0%	18.06	1	0.001
12–18	3 (773)	−0.30	−0.49, −0.12	12.8%			
**Type**							
Open motor skills	5 (419)	−0.72	−0.93, −0.43	26.9%	20.53	1	0.001
Close motor skills	5 (386)	−0.31	−0.57,−0.25	12.4%			
**Studies' quality**							
Scores more than 6 (>6)	6 (884)	−0.33	−0.55, −0.21	6.9%	27.89	1	0.001
Scores less than 6 (≤6)	7 (593)	−0.86	−1.13, −0.39	0.0%			
**Duration**							
More than 12 weeks	6 (336)	−0.36	−0.79, −0.15	0.0%	0.16	1	0.694
Less than 12 weeks	7 (1141)	−0.62	−0.97,−0.26	82.8%			
Composite type							
Multiple-exercise intervention	6 (666)	−0.58	−1.15, −0.37	48.0%	1.47	1	0.257
Sole-exercise intervention	7 (811)	−0.44	−0.83, −0.25	23.2%			
**Frequency**							
1–3 times/week	5 (478)	−0.40	−0.65, −0.15	21.6%	0.10	1	0.953
4–7 times/week	8 (999)	−0.61	−0.61,−0.32	79.6%			
**Exercise session time**							
≤30	4 (295)	−0.82	−1.01, −0.64	59.5%	18.92	1	0.001
>30	9 (1182)	−0.35	−0.47, −0.22	13.1%			

### Meta-Regression Analysis

In order to examine the effect of chronic exercises on inhibitory control and working memory, meta-regression analyses were performed to determine if the variables (age, type, study quality, composite, frequency, time, and duration) influenced the different indices in Tables 7, 8. Regression results showed that open motor skill interventions (β = 0.451645, Q = 1.82, df = 1, *p* = 0.007) and age (β = −0.608123, Q = 2.51, df = 1, *p* = 0.029) were significantly associated with inhibitory control. However, we found no significant relationship between composite interventions and dependent variables on inhibitory control (β = 0.406159, Q = 2.06, df = 1, *p* = 0.064).

**Table 7:** Regression analysis for chronic exercises versus the control group of inhibitory control.

**ES**	**No. of studies/ comparisons**	**Coef. (β)**	**Std. err**.	**95% conf. interval**	**Test for between-groupheterogeneity**		
					**Q-value**	**df (Q)**	***p*-value**
Age^*^	14	−0.608123	0.241830	−1.140387, −0.075859	2.51	1	0.029
Type^**^	15	0.451645	0.137132	0.149819, 0.753470	1.82	1	0.007
Study quality	15	−0.296366	0.229172	−0.800770, 0.208038	0.65	1	0.222
Duration	15	0.006166	0.243023	−0.528724, 0.541055	1.31	1	0.980
Frequency	15	−0.208072	0.225305	−0.703965, 0.287821	0.92	1	0.376
Time	15	−0.378365	0.222389	−1.330843, 0.923009	1.70	1	0.117
Composite	15	0.406159	0.197258	−0.028003, 0.840320	2.06	1	0.064

* shows that the data differ. *p < 0.05,

** *p < 0.01*.

**Table 8:** Regression analysis for chronic exercises vs. the control group of working memory.

**ES**	**No. of studies/ comparisons**	**Coef. (β)**	**Std. err**.	**95% conf. interval**	**Test for between-group heterogeneity**		
					***Q*-value**	**df (Q)**	***p*-value**
Age	12	0.293404	0.263048	−0.225295, 0.683069	4.57	1	0.033
Type^*^	10	−0.375588	0.145919	−0.693518, −0.057659	3.57	1	0.024
Study quality^**^	13	−0.555877	0.105251	−0.785000, −0.326554	5.28	1	0.001
Duration	13	−0.036083	0.333311	−1.29879, −0.090172	0.11	1	0.921
Frequency	13	−0.113366	0.225896	−0.616694, 0.389962	0.50	1	0.627
Time	13	0.303556	0.177347	−0.086782, 0.693894	1.71	1	0.115
Composite	13	−0.235401	0.175217	−0.617166, 0.146365	1.34	1	0.204

* shows that the data differ.*p < 0.05,

** *p < 0.01*.

Regarding the effects of chronic exercises on working memory, both type (β = −0.375588, Q = 3.57, df = 1, *p* = 0.024) and study quality (β =-0.555877, Q = 5.28, df = 1, *p* = 0.001) influenced the ES. Notably, we found that age could significantly moderate the ES of working memory (β = 0.293404, Q = 4.57, df = 1, *p* = 0.033). Additionally, there was no significant relationship between exercise session time and chronic exercises on working memory (β = 0.303556, Q = 1.71, df = 1, *p* = 0.115).

## Discussion

### Summary of Evidence

The present meta-analysis suggests that both acute and chronic exercises may be effective for improving executive functions (e.g., inhibitory control, working memory, and cognitive flexibility) in healthy child and adolescent populations. Moreover, in chronic exercise interventions, working memory was moderated by age, exercise type, and study quality, while only two variables (age and exercise type) played a moderating role in inhibitory control.

### Inhibitory Control

Inhibitory control refers to the conscious inhibition or automatic response in the cognitive process (Wright et al., 2010). The Stroop, Go/no-go, and Flanker tasks are the most commonly used tools to evaluate the performance (reaction time and/or accuracy) of inhibitory control (Chen et al., 2020). The present review suggests that both acute and chronic exercises are beneficial for inhibitory control, with small magnitudes (Xue et al., 2019; Li et al., 2020). The mechanisms of action of acute and chronic exercises on inhibitory control are unclear, but a possible explanation regarding the effects is attributed to the features of exercises; that is, whether acute or chronic exercises are used can cause an individual to need to complete more complex tasks than everyday multitasking, and this operation mode relies on the non-automatic selection of the process during exercises, which facilitates the speed of reaction of inhibition control (Li et al., 2017). Furthermore, a prior study suggested that acute exercises could significantly improve the speed of reaction of inhibitory control in children and adolescents (Ludyga et al., 2016), and a recent study suggested that chronic exercises are equal to the cumulative effect of acute exercises, while the increase in cognitive performance after chronic exercise interventions seems to be reasonable (Pesce, 2012). Therefore, further studies will be needed to identify the relationship between the influence mechanisms of acute exercises and chronic exercises on inhibitory control.

It is common that heterogeneity across studies is present in the meta-analysis, but the impact of acute exercises on the inhibitory control has a small heterogeneity, which is not in agreement with other acute exercise intervention review studies (Moreau and Chou, 2019). This may be due to the fact that our inclusion criteria only included healthy children and adolescents, and evaluation in acute exercise research is carried out immediately after the intervention, which leads to less heterogeneity between different studies. On the contrary, there was considerable heterogeneity regarding the effect of chronic exercises on inhibitory control (*I*^2^ = 68.20%). Meta-regression showed that age and intervention types were moderators of the effect of chronic exercises on inhibitory control, implying that the effect of chronic exercises on inhibitory control improved with age.

Furthermore, the subgroup analysis indicated that, from the age perspective, although 5–12-year-old children and 12–18-year-old adolescents showed positive effects in terms of improving inhibitory control, 5–12-year-old children showed a low inhibitory ability compared with 12–18-year-old adolescents. This finding is in agreement with a previous study (Harnishfeger, 1995) that showed that there is an obvious age trend in the development of inhibition: children between 5 and 10 years of age had a very low inhibition ability, children over 10 years of age began to approach adults, and adults had the strongest inhibition ability (Harnishfeger, 1995). In addition, from the study intervention characteristic perspective, open motor skills can improve inhibition ability significantly more than closed motor skills (*p* = 0.001). This is attributed to the fact that open skills need to respond consistently to changing circumstances. In the process of implementation, information processing methods such as perception, pattern recognition, and decision-making are more prominent than in closed skill exercises, and the level of self-regulation is higher, resulting in significant inhibitory control. A recent meta-analysis demonstrated that the strongest effects emerged from aerobic exercises (motor skills) and cognitively engaging exercises (yoga combined with meditation and stretching) (Vazou et al., 2019). Moreover, a prior study suggested that cognitively engaging physical activity and mentally enriching interventions may promote fundamental changes in the brain that benefit cognition in children (Hillman et al., 2014). These findings indicated that more advanced strategies and cognitive motor skills can contribute to improving inhibitory control for normal child and adolescent populations. Finally, our results showed that medium effect sizes emerged from chronic exercise programs focused on sole exercise interventions (i.e., football, tennis, yoga, wuqinxi). It is important to emphasize that the interpretation of the results from comparisons between sole exercises and multiple exercises of physical activity programs should be conducted with caution due to the small number of studies included in this review.

In addition, the experimental intervention characteristics involving duration, frequency, and exercise session time were not moderators of the effect of chronic exercises on inhibitory control (*p* > 0.05). However, as we found those studies had no long-term follow-up, it remains unclear whether a potential benefit will emerge after a longer period after intervention with chronic exercises on inhibitory control. Therefore, we should not make any definitive claims with respect to composite types and experimental intervention characteristics for chronic exercises in this systematic review.

### Working Memory

Working memory mainly measures the preservation and update of information, and the digit span forward, digit span backward, letter digit span, Tower of London, and N-back task (1-back and 2-back) measurement tools are the most commonly used to evaluate the response time of working memory (Chen et al., 2020). The aggregated results of the present meta-analysis show that acute and chronic exercises are beneficial for working memory in children and adolescents (the magnitude of the effects were statistically significant), and the calculated ESs were −0.72 (acute exercises) and −0.54 (chronic exercises), which have similar medium efficacies for improving working memory. A prior study suggested that acute aerobic exercises have an intensity dose effect on memory in children and adolescents (Rathore and Lom, 2017). Furthermore, early meta regression analysis studies also found that acute aerobic exercises have a promoting effect on memory, and the effect size is greater than that of acute exercises affecting inhibitory control and information processing speed (Lambourne and Tomporowski, 2010). In addition, according to the international physical activity guidelines, chronic exercises are very cognitively beneficial (Schmidt et al., 2015), and a prior study suggested that the cardiorespiratory fitness level of chronic exercises is helpful for the 1-back reaction speed (Luo, 2018). However, regarding the differences between the intervention effects of chronic exercises and acute exercises, the results of a previous study (Rathore and Lom, 2017) are inconsistent with our research results due to the inclusion of older adults, while our criteria include only children and adolescents. Furthermore, some previous studies have suggested that acute exercises have no effect on working memory (Li et al., 2017; De Greeff et al., 2018). Similarly, a prior study suggested that working memory showed no improvement across a range of intervention durations (5–20 min) and intensities (Daly-Smith et al., 2018). The reason for the difference in these results is due to our working memory measurement index extraction method, which only revolved around the processing speed without considering accuracy. The current meta-analysis shows that moderate to high intensity and time (20–30 min) physical activity is effective at enhancing the response time of working memory. Although the mechanisms of action of acute and chronic exercises on working memory are unclear, the contrasting results between chronic and acute exercises offer interesting future directions to explore different mechanisms that govern both intervention types, and need further study.

In addition, our results show that acute exercises were characterized by non-significant heterogeneity (*I*^2^ = 24.4%), which indicates that the 11 studies were not significantly dissimilar from each other, adding further confidence to the result. By contrast, the meta-analysis of the 14 chronic exercise studies revealed a significant heterogeneity (*I*^2^ = 63.4%). Meta-regression showed that study quality and intervention type were moderators of the effect of chronic exercises on working memory. Moderator analysis suggested that a significant difference in working memory between a study quality with a score more than six and a study quality with a score <6 was observed, which implies that a less rigorous study design may consciously affect the results, thus exaggerating the effect of the intervention and resulting in a large effect size. In addition, similarly to the inhibitory control influence mechanism, open motor skills exercises may also significantly improve the reaction of working memory.

Furthermore, experimental intervention characteristics involving duration, frequency, and exercise session time are crucial to investigating the effects of chronic exercise changes in working memory. The moderator analysis indicated that each session time of ≤30 min can improve the response of large ES (−0.82) on working memory; the effect is more significant than for a session time of >30 min. A prior study suggested that the effect of aerobic exercises for 55 min is not as good as for 30 min (Fu and Fan, 2016). The possible reason for this is that an excessively long exercise time cannot induce an appropriate level of arousal and results in fatigue (Moreau and Chou, 2019). Additionally, duration (<12 weeks) and frequency (one to three times) could significantly contribute to moderate ESs on working memory; chronic exercises more than four times per week and for more than 12 weeks did not significantly benefit working memory (*p*_1_ = 0.694, *p*_2_ = 0.953). The reason for this finding is not fully understood, because of the limited experimental design with no long-term follow-up. Therefore, we also should not make any definitive claim with respect to duration and frequency for chronic exercises in working memory. It is worth noting that from the age perspective, our current meta-analysis showed that the intervention effect for children aged 5–12 is greater than that for adolescents aged 12–18 (*p* = 0.001); this is the opposite of the result for the inhibitory control. This may be attributed to the fact that working memory extraction, attention distribution and focus of attention increase significantly at 6–9 years old (Wang et al., 2013), and children aged 5–12 can actively complete tasks in accordance with the teacher's requirements, which is also one of the reasons for their strong subjective initiative.

### Cognitive Flexibility

Cognitive flexibility refers to the ability of individuals to constantly adjust their thoughts and behaviors in order to adapt to changing situations (Hernández et al., 2010). Specifically, when two tasks compete for the same cognitive resource, flexibility is the process of controlling the mutual conversion of these two tasks. The commonly used measurement tasks are as follows: the plus–minus task, number–letter task, more-odd shifting, the local–global task, and the Wisconsin card sorting test (WCST).

Because cognitive flexibility was present across a small number of eligible studies, the present meta-analysis only synthesized WCST and more-odd shifting. With respect to cognitive flexibility, a significant improvement in cognitive flexibility in the present meta-analysis was identified in favor of acute and chronic exercises, but the ES effects of the two types of exercises regarding cognitive flexibility were small (−0.34). It is reasonable that a significant improvement was observed for cognitive flexibility. That is because exercises intervention can change the brain's activation pattern, which specifically manifests as an increased activation of the bilateral upper frontal gyrus, bilateral middle frontal gyrus, and bilateral upper lobules, and an individual is prone to having activated pre-frontal and parietal lobes when exercising cognitive flexibility (Jamadar et al., 2010; Boucard et al., 2012). Therefore, appropriate exercises can improve cognitive flexibility. Although the positive effects of acute and chronic exercises on cognitive flexibility have been shown in the present meta-analysis, it is necessary to explore the literature evidence because of the small number of studies included.

### Study Limitations

This study has a certain number of limitations and deficiencies. (1) There was a limited number of works on cognitive flexibility. Therefore, we could not obtain an accurate result regarding the effect of executive function interventions on children and adolescents. (2) The high-level executive functions, such as decision-making and reasoning, can be understood with a detailed assessment of the dynamics of EF performance, but the studies reviewed in this meta-analysis did not include this information. We believe that future studies should collect, retain, and ideally share these types of data to allow more detailed analyses. (3) Only three articles in this study explained random sequence generation in detail, and no other work mentioned the method of random allocation and hiding. (4) The intervention method in some of the included studies was aerobic exercises, which has not been explained in great detail, and thus we were unable to confirm which skills were involved in aerobic exercises. (5) Most studies had no long-term follow-up, and it remains unclear whether a potential benefit will emerge after a longer period post-intervention of chronic exercises in executive function. (6) The current meta-analysis only made relevant reports on the reaction time; in the future, we also need to report on the effect of physical activity on the accuracy of executive functions.

## Conclusions

The results of the current meta-analysis demonstrate that acute and chronic exercises may have a positive effect on executive function for children and adolescents, especially in terms of working memory. To better understand the effects of acute and chronic exercises on children and adolescents, rigorous study designs are necessary. In addition, the impact of exercise training on cognitive flexibility needs to be further explored. We should explore the impact of long-term physical exercises on cognitive flexibility, which would also provide a reference for improving executive functions through exercises in the future.

## Data Availability Statement

The original contributions presented in the study are included in the article/supplementary material, further inquiries can be directed to the corresponding author.

## Author Contributions

SL, ZL, and YC: conceptualization. SL and ZL: methodology, software, and resources. PC, QY, and YZ: validation. ZK, WL, YZ, and SC: formal analysis. SL, QY, and ZL: investigation and data curation. SL, ZL, and YC: writing—original draft preparation. QY, PC, YZ, ZK, WL, SC, and YC: writing—review and editing. All authors contributed to the article and approved the submitted version.

## Conflict of Interest

The authors declare that the research was conducted in the absence of any commercial or financial relationships that could be construed as a potential conflict of interest.

## References

[B1] Beavan A. Spielmann J. Mayer J. Skorski S. Fransen J. (2020). The rise and fall of executive functions in high-level football players. Psychol. Sport Exerc. 49:101677 10.1016/j.psychsport.2020.101677 32711397

[B2] Benzing V. Heinks T. Eggenberger N. Schmidt M. (2016). Acute cognitively engaging exergame-based physical activity enhances executive functions in adolescents. PLoS ONE 11:e0167501. 10.1371/journal.pone.0167501 28030542 PMC5193332

[B3] Berardelli I. Corigliano V. Hawkins M. Comparelli A. Erbuto D. Pompili M. (2018). Lifestyle interventions and prevention of suicide. Front. Psychiatry 9:567. 10.3389/fpsyt.2018.00567 30459660 PMC6232529

[B4] Best J. R. (2010). Effects of physical activity on children's executive function: contributions of experimental research on aerobic exercise. Develop. Rev. 30, 331–351. 10.1016/j.dr.2010.08.001 21818169 PMC3147174

[B5] Bjoern K. Thomas F. Sabine W. Günter A. (2018). Sport type determines differences in executive functions in elite athletes. Psychol. Sport Exerc. 38, 72–79. 10.1016/j.psychsport.2018.06.002

[B6] Boucard G. K. Albinet C. T. Bugaiska A. Bouquet C. A. Clarys D. Audiffren M. (2012). Impact of physical activity on executive functions in aging: a selective effect on inhibition among old adults. J. Sport Exerc. Psychol. 34, 808–27. 10.1123/jsep.34.6.808 23204360

[B7] Budde H. Niemann C. Wegner M. Koutsandreou F. (2016). Effects of motor versus cardiovascular exercise training on children's working memory. Med. Sci. Sports Exerc. 48, 1145–1152. 10.1249/MSS.0000000000000869 26765631

[B8] Budde H. Voelcker-Rehage C. Pietrassyk-Kendziorra S. Machado S. Ribeiro P. Arafat A. M. (2010). Steroid hormones in the saliva of adolescents after different exercise intensities and their influence on working memory in a school setting. Psychoneuroendocrinology 35, 382–391. 10.1016/j.psyneuen.2009.07.015 19716238

[B9] Byun K. Hyodo K. Suwabe K. Ochi G. Sakairi Y. Kato M.. (2014). Positive effect of acute mild exercise on executive function via arousal-related prefrontal activations: an fNIRS study. Neuroimage 98, 336–345. 10.1016/j.neuroimage.2014.04.067 24799137

[B10] Cardoso H. F. V. (2007). Epiphyseal union at the innominate and lower limb in a modern Portuguese skeletal sample, and age estimation in adolescent and young adult male and female skeletonsandnbsp. Am. J. Phys. Anthropol. 135, 161–170. 10.1002/ajpa.20717 18044694

[B11] Chaddock-Heyman L. Erickson K. I. Voss M. W. Knecht A. M. Pontifex M. B. Castelli D. M.. (2013). The effects of physical activity on functional MRI activation associated with cognitive control in children: a randomized controlled intervention. Front. Hum. Neurosci. 7:72. 10.3389/fnhum.2013.00072 23487583 PMC3594762

[B12] Chen A. Fu L. Zhu L. (2015). Effects of medium-intensity basketball with different durations on children's executive function. J. Capital Ins. Phys. Educ. 27, 223–227. 10.14104/j.cnki.1006-2076.2017.01.015

[B13] Chen A. Li H. Yan J. (2016). Developmental characteristics of executive function and psychosomatic motor intervention in left-behind children. China Spec. Educ. 11, 69–74.

[B14] Chen F.-T. Etnier J. L. Chan K.-H. Chiu P.-K. Hung T.-M. Chang Y.-K. (2020). Effects of exercise training interventions on executive function in older adults: a systematic review and meta-analysis. Sports Med. 50, 1451–1467. 10.1007/s40279-020-01292-x 32447717 PMC7376513

[B15] Chun C. H. Caterina P. Song T. Tsung-Min H. Chang Y.-K. (2015). Failure to identify an acute exercise effect on executive function assessed by the Wisconsin Card Sorting Test. J. Sport Health Sci. 1, 64–72. 10.1016/j.jshs.2014.10.003

[B16] Cooper S. B. Dring K. J. Morris J. G. Sunderland C. Bandelow S. Nevill M. E. (2018). High intensity intermittent games-based activity and adolescents' cognition: moderating effect of physical fitness. BMC Public Health 18:603. 10.1186/s12889-018-5514-6 29739386 PMC5941716

[B17] Daly-Smith A. J. Zwolinsky S. McKenna J. Tomporowski P. D. Defeyter M. A. Manley A. (2018). Systematic review of acute physically active learning and classroom movement breaks on children's physical activity, cognition, academic performance and classroom behaviour: understanding critical design features. Bmj Open Sport Exerc Med. 4:e000341. 10.1136/bmjsem-2018-000341 29629186 PMC5884342

[B18] De Greeff J. W. Bosker R. J. Oosterlaan J. Visscher C. Hartman E. (2018). Effects of physical activity on executive functions, attention and academic performance in preadolescent children: a meta-analysis. J. Sci. Med. Sport 21, 501–507. 10.1016/j.jsams.2017.09.595 29054748

[B19] De Greeff J. W. Hartman E. Mullender-Wijnsma M. J. Bosker R. J. Doolaard S. Visscher C. (2016). Long-term effects of physically active academic lessons on physical fitness and executive functions in primary school children. Health Educ. Res. 2 4:102. 10.1093/her/cyv102 26826113

[B20] Diamond A. (2013). Executive functions. Annu. Rev. Psychol. 64, 135–168. 10.1146/annurev-psych-113011-143750 23020641 PMC4084861

[B21] Egger F. Benzing V. Conzelmann A. Schmidt M. (2019). Boost your brain, while having a break! The effects of long-term cognitively engaging physical activity breaks on children's executive functions and academic achievement. PLoS ONE 14:e0212482. 10.1371/journal.pone.0212482 30840640 PMC6402646

[B22] Egger F. Conzelmann A. Schmidt M. (2018). The effect of acute cognitively engaging physical activity breaks on children's executive functions: too much of a good thing? Psychol. Sport Exerc. 36, 178–186. 10.1016/j.psychsport.2018.02.014

[B23] Ellemberg D. St-Louis-Deschênes M. (2010). The effect of acute physical exercise on cognitive function during development. Psychol. Sport Exerc. 11, 0–126. 10.1016/j.psychsport.2009.09.006

[B24] Ferguson G. D. Aertssen W. F. M. Rameckers E. A. A. Jelsma J. Smits-Engelsman B. C. M. (2014). Physical fitness in children with developmental coordination disorder: measurement matters. Res. Dev. Disabil. 35, 1087–1097. 10.1016/j.ridd.2014.01.031 24582141

[B25] Fisher A. Boyle J. M. E. Paton J. Y. Tomporowski P. Reilly J. J. (2011). Effects of a physical education intervention on cognitive function in young children: randomized controlled pilot study. BMC Pediatr. 11:97. 10.1186/1471-2431-11-97 22034850 PMC3217848

[B26] Flashner B. M. Rifas-Shiman S. L. Oken E. Camargo C. A. Jr. Platts-Mills T. J. Workman L.. (2019). Obesity, sedentary lifestyle, and exhaled nitric oxide in an early adolescent cohort. Pediatr. Pulmonol. 55, 503–509. 10.1002/ppul.24597 31805224 PMC6980304

[B27] Fu J. Fan Y. (2016). Experimental study on the influence of moderate-intensity physical exercise on executive function and academic performance of junior high school students at different times. Sports Sci. 37, 110–116. 10.13598/j.issn1004-4590.2016.06.016

[B28] Goodall J. Fisher C. Hetrick S. Phillips L. Parrish E. M. Allott K. (2018). Neurocognitive functioning in depressed young people: a systematic review and meta-analysis. Neuropsychol. Rev. 28, 216–231. 10.1007/s11065-018-9373-9 29680959

[B29] Gothe N. Pontifex M. B. Hillman C. Mcauley E. (2013). The acute effects of yoga on executive function. J. Phys. Activity Health 10:488. 10.1123/jpah.10.4.488 22820158

[B30] Handoll H. H. G. Howe T. E. Madhok R. (2002). The cochrane database of systematic reviews. Physiotherapy 88, 714–716. 10.1016/S0031-9406(05)60709-2 12076475

[B31] Hanley J. A. Abdissa N. Edwardes M. D. deB. Forrester J. E. (2003). Statistical analysis of correlated data using generalized estimating equations: an orientation. Am. J. Epidemiol. 157, 364–375. 10.1093/aje/kwf215 12578807

[B32] Harnishfeger K. K. (1995). 6–The development of cognitive inhibition: theories, definitions, and research evidence. Interfer. Inhibiti. Cogn. 25, 175–204. 10.1016/B978-012208930-5/50007-6

[B33] Hernández M. Costa A. Fuentes L. J. Vivas A. B. Sebastian Galles N. (2010). The impact of bilingualism on the executive control and orienting networks of attention. Bilingual. Lang. Cogn. 13, 315–325. 10.1017/S1366728909990010

[B34] Hillman C. H. Kamijo K. Scudder M. (2011). A review of chronic and acute physical activity participation on neuroelectric measures of brain health and cognition during childhood. Prevent. Med. 52, 21–28. 10.1016/j.ypmed.2011.01.024 21281669 PMC3094734

[B35] Hillman C. H. Pontifex M. B. Castelli D. M. Khan N. A. Raine L. B. Scudder M. R.. (2014). Effects of the FITKids randomized controlled trial on executive control and brain function. Pediatrics 134, 1063–1071. 10.1542/peds.2013-3219 25266425 PMC4179093

[B36] Jager K. Schmidt M. Conzelmann A. Roebers C. (2015). The effects of qualitatively different acute physical activity interventions in real-world settings on executive functions in preadolescent children. Ment. Health Phys. Act. 9, 1–9. 10.1016/j.mhpa.2015.05.002

[B37] Jamadar S. Hughes M. Fulham W. R. Michie P. T. Karayanidis F. (2010). The spatial and temporal dynamics of anticipatory preparation and response inhibition in task-switching. Neuroimage 51, 432–449. 10.1016/j.neuroimage.2010.01.090 20123028

[B38] Jiang D. (2015). Effects of 8-week moderate intensity football games on executive function development of preschool children. China Sport Sci. Technol. 51, 43–50. 10.16470/j.csst.2015.02.007

[B39] Keita K. Roetzheim R. Gualtieri T. (2011). The effects of an afterschool physical activity program on working memory in preadolescent children. Dev. Sci. 14, 1046–1058. 10.1111/j.1467-7687.2011.01054.x 21884320 PMC3177170

[B40] Kong Z. Sze T.-M. Yu J. J. Loprinzi P. D. Xiao T. Yeung A. S.. (2019). Tai Chi as an alternative exercise to improve physical fitness for children and adolescents with intellectual disability. Int. J. Environ. Res. Public Health 16:1152. 10.3390/ijerph16071152 30935071 PMC6479776

[B41] Koolhaas C. M. van Rooij F. J. A. Kocevska D. Luik A. I. Ikram M. A. Franco O. H. (2019). Objectively measured sedentary time and mental and cognitive health: cross-sectional and longitudinal associations in The Rotterdam Study. Ment. Health Phys. Act. 17, 423–482. 10.1016/j.mhpa.2019.100296

[B42] Kubesch S. Walk L. Spitzer M. Kammer T. Lainburg A. Heim R. (2009). A 30-minute physical education program improves students. Execut Attent. 43, 892–904. 10.1111/j.1751-228X.2009.01076.x

[B43] Kval S. E. Bru E. Brnnick K. Dyrstad S. M. (2017). Does increased physical activity in school affect childrens executive function and aerobic fitness? Scand. J. Med. Sci. Sports 43, 42–72. 10.1111/sms.12856 28207976

[B44] Lambourne K. Tomporowski P. (2010). The effect of exercise-induced arousal on cognitive task performance: a meta-regression analysis. Brain Res. 1341, 12–24. 10.1016/j.brainres.2010.03.091 20381468

[B45] Li J. W. O'Connor H. O'Dwyer N. Orr R. (2017). The effect of acute and chronic exercise on cognitive function and academic performance in adolescents: a systematic review. J. Sci. Med. Sport 20, 841–848. 10.1016/j.jsams.2016.11.025 28185806

[B46] Li L. Zhang J. Cao M. (2020). The effects of chronic physical activity interventions on executive functions in children aged 3-7 years: a meta-analysis. J. Sci. Med. Sport 23, 949–954. 10.1016/j.jsams.2020.03.007 32360243

[B47] Lina Z. (2017). Effects of Aerobic Exercise Intervention on Executive Function and Brain Network Function Connection in Deaf Children. Yangzhou:Yangzhou University.

[B48] Liu S. Xiao T. Yang L. Loprinzi P. D. (2019). Exercise as an alternative approach for treating smartphone addiction: a systematic review and meta-analysis of random controlled trials. Int. J. Environ. Res. Public Health 16, 489–505. 10.3390/ijerph16203912 31618879 PMC6843500

[B49] Loprinzi P. D. Ponce P. Zou L. Li H. (2019). The counteracting effects of exercise on high-fat diet-induced memory impairment: a systematic review. Brain Sci. 9:145. 10.3390/brainsci9060145 31226771 PMC6627483

[B50] Ludyga S. Gerber M. Brand S. Holsboer-Trachsler E. Puhse U. (2016). Acute effects of moderate aerobic exercise on specific aspects of executive function in different age and fitness groups: a meta-analysis. Psychophysiology 53, 1611–1626. 10.1111/psyp.12736 27556572

[B51] Ludyga S. Gerber M. Herrmann C. Brand S. Pühse U. (2017). Chronic effects of exercise implemented during school-break time on neurophysiological indices of inhibitory control in adolescents. Trends Neuro. Educ. 10, 1–7. 10.1016/j.tine.2017.11.00129358034

[B52] Ludyga S. Gerber M. Kamijo K. Brand S. Pühse U. (2018). The effects of a school-based exercise program on neurophysiological indices of working memory operations in adolescents. J. Sci. Med. Sport 32, 178-189. 10.1016/j.jsams.2018.01.001 29358034

[B53] Luo J. (2018). Review on the influence of physical exercise on working memory. J. Shandong Univ. Phys. Educ. 34, 70–77.

[B54] Macedo L. G. Elkins M. R. Maher C. G. Moseley A. M. Herbert R. D. Sherrington C. (2010). There was evidence of convergent and construct validity of Physiotherapy Evidence Database quality scale for physiotherapy trials. J. Clin. Epidemiol. 63, 920–925. 10.1016/j.jclinepi.2009.10.005 20171839

[B55] Maher C. G. Sherrington C. Herbert R. D. (2003). Reliability of the PEDro scale for rating quality of randomized controlled trials. Phys. Ther. 83, 713–721. 10.1093/ptj/83.8.713 12882612

[B56] Masley S. Roetzheim R. Gualtieri T. (2009). Aerobic exercise enhances cognitive flexibility. J. Clin. Psychol. Med. Sett. 16:186. 10.1007/s10880-009-9159-6 19330430

[B57] Mcminn D. (2012). Re: Effectiveness of intervention on physical activity of children: systematic review and meta-analysis of controlled trials with objectively measured outcomes (EarlyBird 54). BMJ Clin. Res. 345:e5888 10.1136/bmj.e5888 23044984

[B58] McSween M.-P. Coombes J. S. MacKay C. P. Rodriguez A. D. Erickson K. I. Copland D. A.. (2019). The immediate effects of acute aerobic exercise on cognition in healthy older adults: a systematic review. Sports Med. 49, 67–82. 10.1007/s40279-018-01039-9 30560422

[B59] Moreau D. Chou E. (2019). The acute effect of high-intensity exercise on executive function: a meta-analysis. Perspect. Psychol. Sci. 14, 734–764. 10.1177/1745691619850568 31365839

[B60] Nie X. (2019). An experimental study on the Effect of Wuqinxi Exercise on executive function of junior high school students. J. Shandong Normal Univ. 3:32.

[B61] Padulo J. Bragazzi N. L. De Giorgio A. Grgantov Z. Prato S. Ardigò L. P. (2019). The effect of physical activity on cognitive performance in an italian elementary school: insights from a pilot study using structural equation modeling. Front. Physiol. 10:202. 10.3389/fphys.2019.00202 30890960 PMC6412095

[B62] Park S. Etnier J. L. (2019). Beneficial effects of acute exercise on executive function in adolescents. J. Phys. Activ. Health. 16, 1–7. 10.1123/jpah.2018-0219 31010372

[B63] Pate R. R. (2015). Acute effects of classroom exercise breaks on executive function and math performance: a dose–response study. Res. Q. Exerc. Sport 32, 212–221. 10.1080/02701367.2015.1039892 26009945

[B64] Pesce C. (2012). Shifting the focus from quantitative to qualitative exercise characteristics in exercise and cognition research. J. Sport Exerc. Psychol. 34, 766–786. 10.1123/jsep.34.6.766 23204358

[B65] Pindus D. M. Drollette E. S. Raine L. B. Kao S.-C. Khan N. Westfall D. R.. (2019). Moving fast, thinking fast: the relations of physical activity levels and bouts to neuroelectric indices of inhibitory control in preadolescents. J. Sport Health Sci. 8, 301–314. 10.1016/j.jshs.2019.02.003 31333883 PMC6620425

[B66] Press S. (2009). Stata Longitudinal Data/Panel Data Reference Manual (Release 11). Drive College Station, TX: Stata Press.

[B67] Purohit S. P. Pradhan B. (2016). Effect of yoga program on executive functions of adolescents dwelling in an orphan home: a randomized controlled study. J. Trad. Complem. Med. 7, 99–105. 10.1016/j.jtcme.2016.03.001 28053894 PMC5198826

[B68] Qi F. Kong Z. Xiao T. Leong K. Zschorlich V. R. Zou L. (2019). Effects of combined training on physical fitness and anthropometric measures among boys aged 8 to 12 years in the physical education setting. Sustainability 11:1219 10.3390/su11051219

[B69] Rathore A. Lom B. (2017). The effects of chronic and acute physical activity on working memory performance in healthy participants: a systematic review with meta-analysis of randomized controlled trials. Syst. Rev. 6:124. 10.1186/s13643-017-0514-7 28666470 PMC5493123

[B70] Rey-Lopez J. (2008). Sedentary behaviour and obesity development in children and adolescents. Nutr. Metab. Cardiovasc. Dis. 3, 718–734. 10.1016/j.numecd.2007.07.008 18083016

[B71] Riquelme I. Arnould C. Hatem S. M. Bleyenheuft Y. (2019). The two-arm coordination test: maturation of bimanual coordination in typically developing children and deficits in children with unilateral cerebral palsy. Dev. Neurorehabil. 22, 312–320. 10.1080/17518423.2018.1498552 30024779

[B72] Rocha M. S. Yaruss J. S. Rato J. R. (2019). Temperament, executive functioning, and anxiety in school-age children who stutter. Front. Psychol. 10:2244. 10.3389/fpsyg.2019.02244 31636587 PMC6788391

[B73] Schmidt M. JäGer K. Egger F. Roebers C. M. Conzelmann A. (2015). Cognitively engaging chronic physical activity, but not aerobic exercise, affects executive functions in primary school children: a group-randomized controlled trial. J. Sport Exerc. Psychol. 37:575 10.1123/jsep.2015-0069 26866766

[B74] Sember V. Jurak G. Kovac M. Morrison S. A. Starc G. (2020). Children's physical activity, academic performance, and cognitive functioning: a systematic review and meta-analysis. Front. Public Health 8:307. 10.3389/fpubh.2020.00307 32760689 PMC7372103

[B75] Shamseer L. Moher D. Clarke M. Ghersi D. Liberati A. Petticrew M. (2015). Preferred reporting items for systematic review and meta-analysis protocols (PRISMA-P) 2015: elaboration and explanation. Bmj 349, 489–499. 10.1136/bmj.g7647 25555855

[B76] Sisson S. B. Church T. S. Martin C. K. Tudor-Locke C. Smith S. R. Bouchard C.. (2009). Profiles of sedentary behavior in children and adolescents: The US National Health and Nutrition Examination Survey, 2001–2006. Int. J. Pediatr. Obes. 4, 353–359. 10.1080/17477160902934777 19922052 PMC2891818

[B77] Stroth S. Kubesch S. Dieterle K. Ruchsow M. Heim R. Kiefer M. (2009). Physical fitness, but not acute exercise modulates event-related potential indices for executive control in healthy adolescents. Brain Res. 1269, 114–124. 10.1016/j.brainres.2009.02.073 19285042

[B78] Taddei F. Bultrini A. Spinelli D. Russo F. D. (2012). Neural correlates of attentional and executive processing in middle-age fencers. Med. Sci. Sports Exerc. 44, 1057–1066. 10.1249/MSS.0b013e31824529c2 22157879

[B79] Tarp J. Domazet S. L. Froberg K. Hillman C. H. Andersen L. B. Bugge A. (2016). Effectiveness of a school-based physical activity intervention on cognitive performance in Danish Adolescents: LCoMotion—learning, cognition and motion – a cluster randomized controlled trial. PLoS ONE 11:e0158087. 10.1371/journal.pone.0158087 27341346 PMC4920412

[B80] Telles S. Singh N. Bhardwaj A. K. Kumar A. Balkrishna A. (2013). Effect of yoga or physical exercise on physical, cognitive and emotional measures in children: a randomized controlled trial. Child Adolesc. Psychiatry Ment. Health 7:37 10.1186/1753-2000-7-37 24199742 PMC3826528

[B81] Thivel D. Tremblay A. Genin P. M. Panahi S. Rivière D. Duclos M. (2018). Physical activity, inactivity, and sedentary behaviors: definitions and implications in occupational health. Front. Public Health 6:288. 10.3389/fpubh.2018.00288 30345266 PMC6182813

[B82] Torrens-Burton A. Basoudan N. Bayer A. J. Tales A. (2017). Perception and reality of cognitive function: information processing speed, perceived memory function, and perceived task difficulty in older adults. J. Alzheimers Dis. 60, 1601–1609. 10.3233/JAD-170599 28984584 PMC5676981

[B83] Tremblay M. S. LeBlanc A. G. Janssen I. Kho M. E. Hicks A. Murumets K.. (2011). Canadian sedentary behaviour guidelines for children and youth. Appl. Physiol. Nutr. Metabol. 36, 59–64. 10.1139/H11-012 21326378

[B84] Vazou S. Pesce C. Lakes K. Smiley-Oyen A. (2019). More than one road leads to Rome: a narrative review and meta-analysis of physical activity intervention effects on cognition in youth. Int. J. Sport Exerc. Psychol. 17, 153–178. 10.1080/1612197X.2016.1223423 31289454 PMC6615761

[B85] Vera V. D. B. Saliasi E. De Groot R. H. M. Chinapaw M. J. M. Singh A. S. (2019). Improving cognitive performance of 9–12 years old children: just dance? A Randomized Controlled Trial. Front. Psychol. 10:174. 10.3389/fpsyg.2019.00174 30787899 PMC6372522

[B86] Vera V. D. B. Saliasi E. Jolles J. De Groot R. H. M. Chinapaw M. J. M. Singh A. S. (2018). Exercise of varying durations: no acute effects on cognitive performance in adolescents. Front. Neurosci. 12:672. 10.3389/fnins.2018.00672 30319345 PMC6171199

[B87] Wang X. Li Y. Fan H. (2019). The associations between screen time-based sedentary behavior and depression: a systematic review and meta-analysis. BMC Public Health 19, 32–58. 10.1186/s12889-019-7904-9 31727052 PMC6857327

[B88] Wang X. Ma J. Sun X. Sun Z. (2013). Development of working memory in children aged 6-9 years. Psychol. Sci. 36, 92–97. 10.16719/j.cnki.1671-6981.2013.01.029

[B89] Wang Z. (2017). Effect of 8-week tennis game on executive control function of preschool children. Capital Ins. Phys Educ. (Beijing).

[B90] Weng T. B. Pierce G. L. Darling W. G. Voss M. W. (2014). Differential effects of acute exercise on distinct aspects of executive function. Med. Sci. Sports Exerc. 47, 1460–1469. 10.1249/MSS.0000000000000542 25304335

[B91] Willoughby M. T. Blair C. B. Wirth R. J. Greenberg M. (2012). The measurement of executive function at age 5: psychometric properties and relationship to academic achievement. Psychol. Assess 24, 226–239. 10.1037/a0025361 21966934 PMC3315058

[B92] Wright I. Waterman M. Prescott H. Murdoch-Eaton D. (2010). A new Stroop-like measure of inhibitory function development: typical developmental trends. J. Child Psychol. Psychiatry 44, 561–575. 10.1111/1469-7610.00145 12751848

[B93] Xin L. (2012). Effects of Short-Term Moderate-Intensity Aerobic Exercise on Executive Function of Female College Students. Shanghai: East China Normal University.

[B94] Xue Y. Yang Y. Huang T. (2019). Effects of chronic exercise interventions on executive function among children and adolescents: a systematic review with meta-analysis. Br. J. Sports Med. 53:1397. 10.1136/bjsports-2018-099825 30737201

[B95] Yan Jun M. S. Chen A. (2013). Experimental study on the effect of different duration aerobics exercises on executive function of college girls. Sport Sci. 33, 88–91. 10.13297/j.cnki.issn1005-0000.2014.04.015

[B96] Yan J. Wu Y. Chen A. (2014). Effects of short-term, medium-intensity and different types of exercise on executive function of primary school students. Sport Sci. 35, 94–100. 10.13598/j.issn1004-4590.2012.06.016

[B97] Yin H. Pan J. Lai Y. (2018). Development and empirical study of exercise intervention programs to improve brain executive function of pupils with different types of learning difficulties. J. Wuhan Ins. Phys. Educ. 52, 78–89.

[B98] Zhang Y. Alonso-Coello P. Guyatt G. H. Yepes-Nunez J. J. Akl E. A. Hazlewood G.. (2019). GRADE Guidelines: 19. Assessing the certainty of evidence in the importance of outcomes or values and preferences-Risk of bias and indirectness. J. Clin. Epidemiol. 111, 94–104. 10.1016/j.jclinepi.2018.01.013 29452223

[B99] Zhu F. (2015). Research on the Improvement of college students' self-control ability and brain processing characteristics by Physical *exercise*. PhD, Shanghai Institute of Physical Education, Shanghai, China.

[B100] Zou L. Loprinzi P. D. Yeung A. S. Zeng N. Huang T. (2019). The beneficial effects of mind-body exercises for people with mild cognitive impairment: a systematic review with meta-analysis. Arch. Phys. Med. Rehabil. 100, 1556–1573. 10.1016/j.apmr.2019.03.009 30986409

[B101] Zou L. Sasaki J. E. Zeng N. Wang C. Sun L. (2018). A systematic review with meta-analysis of mindful exercises on rehabilitative outcomes among poststroke patients. Arch. Phys. Med. Rehabil. 99, 2355–2364. 10.1016/j.apmr.2018.04.010 29738744

